# Precision dosimetry in yttrium-90 radioembolization through CT imaging of radiopaque microspheres in a rabbit liver model

**DOI:** 10.1186/s40658-022-00447-1

**Published:** 2022-03-21

**Authors:** E. Courtney Henry, Matthew Strugari, George Mawko, Kimberly Brewer, David Liu, Andrew C. Gordon, Jeffrey N. Bryan, Charles Maitz, James J. Karnia, Robert Abraham, S. Cheenu Kappadath, Alasdair Syme

**Affiliations:** 1 Department of Physics and Atmospheric Science, Dalhousie University, Halifax, NS Canada; 2 Biomedical Translational Imaging Centre, Halifax, NS Canada; 3 Department of Medical Physics, Nova Scotia Health Authority, Halifax, NS Canada; 4 Department of Radiation Oncology, Dalhousie University, Halifax, NS Canada; 5 Department of Diagnostic Radiology, Dalhousie University, Halifax, NS Canada; 6 Department of Biomedical Engineering, Dalhousie University, Halifax, NS Canada; 7 ABK Biomedical Inc., Halifax, NS Canada; 8 School of Biomedical Engineering, University of British Columbia, Vancouver, BC Canada; 9 Department of Radiology, Northwestern University, Chicago, IL USA; 10 Department of Veterinary Medicine and Surgery, University of Missouri, Columbia, MO USA; 11 Department of Imaging Physics, University of Texas MD Anderson Cancer Centre, Houston, TX USA

**Keywords:** Radioembolization, Yttrium-90, Radiopacity, Microsphere, Dosimetry, PET, CT

## Abstract

**Purpose:**

To perform precision dosimetry in yttrium-90 radioembolization through CT imaging of radiopaque microspheres in a rabbit liver model and to compare extracted dose metrics to those produced from conventional PET-based dosimetry.

**Materials and methods:**

A CT calibration phantom was designed containing posts with nominal microsphere concentrations of 0.5 mg/mL, 5.0 mg/mL, and 25.0 mg/mL. The mean Hounsfield unit was extracted from the post volumes to generate a calibration curve to relate Hounsfield units to microsphere concentration. A nominal bolus of 40 mg of microspheres was administered to the livers of eight rabbits, followed by PET/CT imaging. A CT-based activity distribution was calculated through the application of the calibration curve to the CT liver volume. Post-treatment dosimetry was performed through the convolution of yttrium-90 dose-voxel kernels and the PET- and CT-based cumulated activity distributions. The mean dose to the liver in PET- and CT-based dose distributions was compared through linear regression, ANOVA, and Bland–Altman analysis.

**Results:**

A linear least-squares fit to the average Hounsfield unit and microsphere concentration data from the calibration phantom confirmed a strong correlation (*r*^2^ > 0.999) with a slope of 14.13 HU/mg/mL. A poor correlation was found between the mean dose derived from CT and PET (*r*^2^ = 0.374), while the ANOVA analysis revealed statistically significant differences (*p* < 10^−12^) between the MIRD-derived mean dose and the PET- and CT-derived mean dose. Bland–Altman analysis predicted an offset of 15.0 Gy between the mean dose in CT and PET. The dose within the liver was shown to be more heterogeneous in CT than in PET with an average coefficient of variation equal to 1.99 and 1.02, respectively.

**Conclusion:**

The benefits of a CT-based approach to post-treatment dosimetry in yttrium-90 radioembolization include improved visualization of the dose distribution, reduced partial volume effects, a better representation of dose heterogeneity, and the mitigation of respiratory motion effects. Post-treatment CT imaging of radiopaque microspheres in yttrium-90 radioembolization provides the means to perform precision dosimetry and extract accurate dose metrics used to refine the understanding of the dose–response relationship, which could ultimately improve future patient outcomes.

## Introduction

World-wide, hepatocellular carcinoma (HCC) is the fifth most frequently diagnosed cancer in men and the ninth in women with more than 840,000 new cases each year [1]. It is responsible for the third most frequent cause of cancer-related deaths and is one of a small number of cancers with a growing rate of incidence, particularly in Europe and North America where there have been historically low rates of incidence [1, 2]. Although liver transplant or surgical resection is considered the most effective treatment, 95% of patients are diagnosed after the disease has progressed beyond the point where these treatments are an option [3–6]. For many of these patients, embolic therapies may prolong life expectancy [5].

Yttrium-90 (^90^Y) radioembolization (RE)—indicated for primary and metastatic liver cancer—is a radiation-based embolic therapy that has been integrated into clinical practice for more than 20 years [7]. In RE, ^90^Y-labelled microspheres are administered into the hepatic arterial vasculature to preferentially target liver tumours while sparing the surrounding liver parenchyma. This preferential uptake is achieved by exploiting the process of tumour tissue angiogenesis, resulting in tumoural vascular inflow derived exclusively from the hepatic artery, while the healthy liver parenchyma receives approximately 80% of its vascular inflow from the portal vein [8]. The liver’s dual blood supply is exploited to overcome the inherent limitation of external beam radiotherapy—the irradiation of healthy tissue while treating the intended target.

The two commercially available microspheres with FDA and CE approval are TheraSphere® glass microspheres (Boston Scientific Corp., Marlborough, MA, USA) and resin-based SIR-Spheres® microspheres (Sirtex Medical Inc., Woburn, MA, USA). As a therapeutic agent, both microspheres employ ^90^Y—a pure β^−^ emitter that decays to zirconium-90 (^90^Zr) with a physical half-life of 2.67 days (64.1 h). Approximately 95% of ^90^Y activity will have decayed after 11.5 days [9]. Maximum and average β^−^ energies are 2.28 MeV and 0.93 MeV, respectively, corresponding to a continuous slowing down approximation (CSDA) range in water of 11.0 mm and 2.4 mm. The therapeutic range $X_{90}$ in water is 5.4 mm, where $X_{90}$ is defined as the radius of a sphere containing 90% of the absorbed dose [10].

With this localized energy deposition, the microsphere spatial distribution within the tumour plays a critical role in determining the absorbed dose. It has been previously demonstrated that microsphere distributions observed in ex vivo tissue samples can be highly heterogeneous with a wide range of microsphere cluster sizes [11–14]. Furthermore, microspheres preferentially accumulate in the tumour’s periphery and tend to remain localized within the portal tracts of the liver’s vasculature [11, 15]. These combined effects result in a highly heterogeneous dose distribution. To demonstrate, Roberson et al. estimated that in a small tumour nodule, the minimum tumour dose was less than half of the average dose [14]. A study performed by Kennedy et al. found that a TheraSphere administration intended to deliver a nominal dose of 150 Gy actually delivered doses between 100 and 8000 Gy, although only a small fraction of the volume received a dose exceeding 1000 Gy [12]. Cremonesi et al. later demonstrated the dose rate around a point source of ^90^Y varied by approximately five orders of magnitude over a distance of only 2 mm [16].

To facilitate patient-specific estimates of the absorbed dose, post-treatment ^90^Y PET and bremsstrahlung SPECT (bSPECT) imaging can approximate the ^90^Y activity distribution. The signal generated in bSPECT images arises from bremsstrahlung produced as ^90^Y β^−^ particles traverse the soft tissue and interact with atomic nuclei. In the case of PET, the 511 keV annihilation photons are generated in an electron–positron annihilation event and subsequently detected in coincidence. The positron is produced through internal pair production in the decay of ^90^Y to stable ^90^Zr, although this decay pathway is infrequent with a branching ratio of only 3.186 ± 0.047 × 10^−5^ [17]. Provided with a SPECT- or PET-based activity distribution, voxel-based dosimetry formalisms can be employed to calculate the absorbed dose distribution [18]. Accumulated evidence from retrospective dose–response studies in ^90^Y RE for the treatment of HCC with TheraSphere microspheres suggests a positive correlation between absorbed dose and tumour response, but the range of reported dose thresholds varies by a full order of magnitude [19]. Variance in the data may be attributed to tumour size, follow-up time, response assessment criteria, and the heterogeneity of study design; however, the poor spatial resolution of ^90^Y PET and bSPECT arguably poses the most significant limitation to accurately quantifying dose thresholds for the prediction of toxicity, response, and survival in ^90^Y RE [20]. The spatial resolution, as measured by the full width at half maximum (FWHM**),** is reported to lie between 5.0 and 10.0 mm in ^90^Y PET imaging and between 7.0 and 30.0 mm in bSPECT imaging [21–23]. In either case, the resolution is insufficient to accurately estimate the true ^90^Y activity distribution as the FWHM is consistently greater than the average ^90^Y β^−^ emission range (2.4 mm) and orders of magnitude greater than the distance scale (microns) over which changes in microsphere concentration take place [12, 13]. This limitation results in the blurring of the true ^90^Y activity distribution, which can diminish variations in the absorbed dose and incorrectly yield a more homogeneous dose distribution. Consequently, there exists an unmet clinical need to provide substantially higher spatial resolution imaging of microsphere distributions to facilitate high-accuracy, high-precision dosimetry in ^90^Y RE.

CT-based imaging can provide significantly enhanced spatial resolution imaging relative to PET and bSPECT imaging, but CT-based evaluations of commercial microspheres are not performed as they lack sufficient radiopacity for their visualization to be of clinical use [24, 25]. Fortunately, a preclinical radiopaque microsphere product called Eye90 microspheres™ has recently been developed (ABK Biomedical Inc., Halifax, NS, Canada). By virtue of the high effective atomic numbers of the compounds within the material composition of these microspheres, they provide substantial radiopacity which permits high-resolution CT imaging to visualize the microspheres’ spatial distribution. This accurate portrayal of the microsphere’s spatial distribution, and hence ^90^Y activity distribution, enables precise CT-based dosimetry [26]. Furthermore, due to the fast scan time of CT relative to PET, uncertainties attributed to respiratory motion during the PET image acquisition can be effectively eliminated in CT as acquisitions can be performed with a breath hold technique.

Beyond the mean absorbed dose provided by PET and bSPECT dosimetry, CT-based dosimetry can provide accurate measures of dose-volume metrics, such as $D_{70}$—the minimum dose absorbed by at least 70% of a target volume. Previous work has shown that the poor spatial resolution of PET and bSPECT imaging may provide inaccurate estimates of $D_{70}$ as this metric has been shown to correlate with the voxel size of the imaging modality [26].

The purpose of this study is to perform precision dosimetry in ^90^Y RE through CT imaging of radiopaque microspheres in a rabbit liver model and compare extracted dose metrics to those produced from PET-based dosimetry. This is the first study to provide an in vivo comparison of PET- and CT-based dosimetry in ^90^Y RE.

## Materials and methods

Numerical values are reported as mean ± standard deviation [minimum; maximum], unless otherwise stated.

### Radiopaque microspheres

The Eye90 microspheres (Eye90) were composed of a proprietary, radiopaque glass composition and were similar in density and size to TheraSphere microspheres (20–30 µm diameter, *ρ* = 3.4 g/cm^3^). The ^90^Y in Eye90 was produced through thermal neutron absorption of yttrium-89 embedded within the microsphere’s glass matrix. A nominal bolus of 40 mg (~ 981,000 microspheres) was measured for administration to each rabbit. The average microsphere activity $A_{{{\text{MS}}}}$ at the time of administration was 156 ± 18 Bq [142; 182].

### CT calibration phantom

**Fig. 1:**
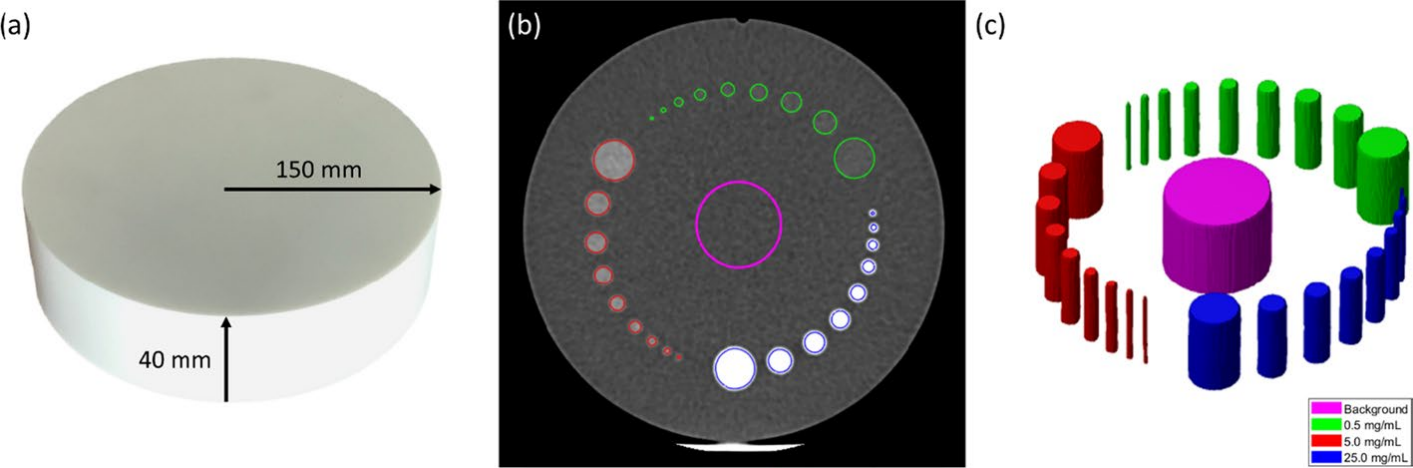
**a** Image of the calibration phantom overlaid with physical dimensions. **b** Axial CT slice [− 100 to 200 HU] of the calibration phantom with segmented structures for a background region (magenta) and three microsphere concentrations: 0.5 mg/mL (green), 5.0 mg/mL (red), and 25.0 mg/mL (blue). **c** Segmented structures in the calibration phantom

It has been previously demonstrated that a linear relationship, defined by Eq. 1, exists between CT Hounsfield units HU and radiopaque microsphere concentration MS_con_ over a clinically relevant range of values,

1 $${\text{HU}} = m_{{{\text{cal}}}} \cdot {\text{MS}}_{{{\text{con}}}} + b_{{{\text{cal}}}}$$

where $m_{{{\text{cal}}}}$ and $b_{{{\text{cal}}}}$ are the slope and intercept (defined below) of the calibration curve, respectively [27]. Based on this relationship, a calibration phantom was designed to contain cylindrical posts composed of a tissue-equivalent resin ($Z_{{{\text{eff}}}}$ = 6.45, $\rho$ = 1.03 g/cm^3^) and infused with Eye90 in nominal concentrations of 0.5 mg/mL, 5.0 mg/mL, 25.0 mg/mL. The calibration posts were produced by mixing the microspheres with a viscous resin then immediately pouring the mixture into a cylindrical mould. At room temperature, the resin-microsphere mixture cured within two minutes, allowing the microspheres to remain in suspension within the mixture. Evaluation of each post with CT imaging (data not shown) revealed excellent uniformity across the entire post length. There were nine posts per microsphere concentration with a post length of 40 mm and varying diameters ranging from 2 to 9 mm in 1 mm increments, with an additional post having a diameter of 15 mm. The central axis of all posts was placed equidistant (100 mm) from the central longitudinal axis and was embedded in the resin background material having a radius of 150 mm, as shown in Fig. 1a.

This phantom was imaged with a clinical CT (Celesteion™ PET/CT, Canon Medical Systems, Ōtawara, Japan) using a tube potential of 100 kVp and an exposure of 270 mAs. Images were reconstructed with filtered back projection in a 16.2 cm field of view (FOV) having voxel dimensions of 0.313 mm × 0.313 mm × 2.000 mm. An axial CT slice of the phantom is shown in Fig. 1b and is displayed with a voxel intensity range of − 100 to 200 HU. Within the MIM Software platform v6.9.4 (MIM Software Inc., Cleveland, OH, USA), structures were created for all 27 posts based on the known geometry of the phantom. Segmented post structures were reduced by a 1 mm radial margin and 5 mm longitudinal margin to reduce partial volume effects between the background-post and background-air interfaces, respectively. The 2 mm-diameter post segmentations were not reduced by the 1 mm radial margin as this would eliminate the structures entirely. Instead, they were reduced to a 1 mm-diameter cylinder centred on the post’s central longitudinal axis. An additional cylindrical structure with a 30 mm diameter was segmented in the centre of the background region to quantify the intensity of a uniform volume void of microspheres. All segmented structures within the calibration phantom are shown in Fig. 1c.

The mean HU was extracted from each structure, and a calibration curve based on Eq. 1 was determined through a linear least-squares fit of the HU and MS_con_ data. The slope *m*_cal_ was extracted from the fit, while the intercept *b*_cal_ was calculated independently for each rabbit according to Eq. 2.

2 $$b_{{{\text{cal}}}} = \mu_{{{\text{bkg}}}} + 1.645_{{{\text{bkg}}}}$$

Here $\mu_{{{\text{bkg}}}}$ and $\sigma_{{{\text{bkg}}}}$ are the mean and standard deviation, respectively, of CT voxel values in a non-embolized background region $L_{{{\text{bkg}}}}$ within each rabbit liver to account for HU variations in the liver parenchyma between rabbits. The factor 1.645 is the Z-score for a one-sided standard normal distribution with a false positive detection rate of $\alpha = 0.05$. As the voxel values within bkg were normally distributed, voxels with ${\text{HU}} > b_{{{\text{cal}}}}$ have a 95% probability of containing Eye90.

To produce a voxelized CT-based ^90^Y activity distribution *A*_CT_ with units of Bq, Eq. 1 was solved for ${\text{MS}}_{{{\text{con}}}}$ and multiplied by three scalar factors: the number of microspheres per milligram ${\text{MS}}_{{{\text{mg}}}}$, the microsphere specific activity $A_{{{\text{MS}}}}$ measured at the time of administration, and the CT voxel volume $V_{{{\text{CT}}}}$, as shown in Eq. 3.

3 $$A_{{{\text{CT}}}} = {\text{MS}}_{{{\text{con}}}} \cdot [{\text{MS}}_{{{\text{mg}}}} \cdot A_{{{\text{MS}}}} \cdot V_{{{\text{CT}}}} ]$$

The number of microspheres per milligram was determined by measuring the diameter of a group of microspheres (*n* = 28) through microscopy and calculating the average microsphere volume. Given the volume and a microsphere density of 3.4 g/cm^3^, the microsphere mass was determined to be 4.09 ± 0.09 × 10^−5^ mg [3.96 × 10^−5^; 4.33 × 10^−5^]. Therefore, the average number of microspheres per milligram MS_mg_ is equal to 24,460 ± 542 [23,111; 25,227].

Theoretically, the administered ^90^Y activity $A_{0}$ should be recovered by summing the voxelized activity distribution *A*_CT_ over the segmented liver volume $L$. A recovery coefficient RC_CT_ was defined as the ratio of this sum to $A_{0}$, expressed as a percentage and shown in Eq. 4.

4 $${\text{RC}}_{{{\text{CT}}}} = 100 \cdot \left[ {\frac{{\mathop \sum \nolimits_{L} A_{{{\text{CT}}}} }}{{A_{0} }}} \right]$$

For comparison, Eq. 4 is applied to the PET-derived activity distribution *A*_PET_ for two structures: the liver volume $L$ and an extended liver volume $L_{{{\text{shell}}}}$, defined as $L$ plus a 1 cm isotropic margin. The corresponding recovery coefficients are ${\text{RC}}_{{{\text{PET}}}}$ and ${\text{RC}}_{{{\text{PET}}}}^{{{\text{shell}}}}$, respectively.

### Rabbit liver model

The University of Missouri Animal Care and Use Committee approved the animal protocol (#9786) whose data were analysed for this study. Eight White New Zealand rabbits were included in this study (5 males, 3 females) weighing an average of 3.3 ± 0.2 kg [3.0; 3.5]. Rabbits are subsequently referred to as R01 through R08.

Prior to administration, each rabbit was induced with ketamine and dexmedetomidine then maintained on isoflurane and oxygen by mask. Eye90 was administered into either the left or proper hepatic arteries of the liver via a 2.4 Fr Progreat microcatheter. The average whole liver volume was 79 ± 11 mL [65; 97]. The ^90^Y activity in Eye90 was measured with a Ludlum Model 3 survey meter (Ludlum Measurements Inc., Sweetwater, TX, USA) following neutron activation. Decay correction was applied to the time of microsphere administration. The average ^90^Y activity at the time of administration was found to be 153.8 ± 18.0 MBq [140.0; 180.0]. Following administration, residual ^90^Y activity in the microsphere vial was measured using the survey meter with the same geometry used to assay the activity post-activation. The residual ^90^Y activity within the microsphere administration lines was also measured following their placement in a Nalgene container. To account for geometric variations, four measurements were acquired at 90° intervals as the container was rotated through 360°. Measurements were then averaged and added to the residual activity in the microsphere vial to determine a total residual activity of 8.9 ± 2.0 MBq [6.0; 12.9]. Therefore, the average administered activity $A_{0}$ was 144.2 ± 17.4 MBq [128.1; 171.0]. The lung shunt fraction was expected to be negligible based on results from pathologic studies of a rabbit VX2 liver tumour model following the administration of iron oxide microspheres [28]. In this study, both intra-procedural fluoroscopic imaging and post-procedural PET imaging verified microsphere deposition only within the liver volume $L$.

### Post-treatment imaging

Following microsphere administration, each rabbit was imaged with a time-of-flight (TOF) PET/CT scanner (Celesteion™ PET/CT, Canon Medical Systems, Ōtawara, Japan) while under anaesthesia. The radioisotope ^90^Y was selected for the PET acquisition. Data were acquired using four overlapping bed positions with seven minutes/position. The lower and upper energy level discriminators were set to 435 keV and 650 keV, respectively. Prior to reconstruction, sinograms were corrected for scatter using a model-based scatter correction method [29] and for attenuation using CT image data [30]. Random coincidences were corrected for using a delayed coincidence approach [31]. Images were reconstructed in a 128 × 128 × 240 matrix using a 26.0 cm transaxial FOV with isotropic voxel sizes of 2.039 mm × 2.039 mm × 2.039 mm. The reconstruction algorithm employed in this study was the ordered subset expectation maximization algorithm with three iterations and ten subsets [32]. Post-filtering of reconstructed images was performed with a 4 mm FWHM Gaussian filter to reduce image noise.

**Fig. 2:**
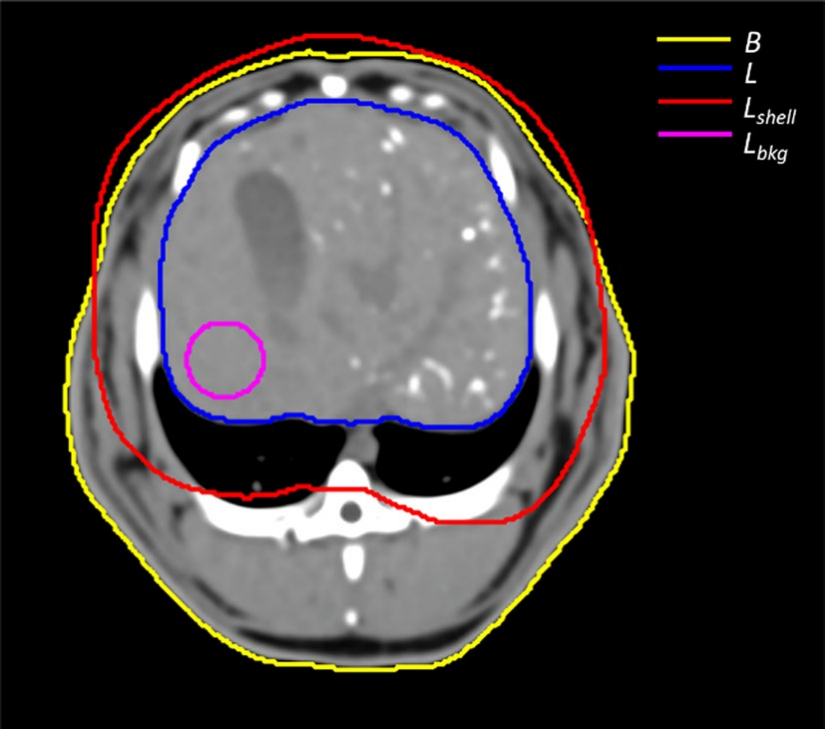
Baseline unenhanced axial CT slice [− 100 to 200 HU] of R03 following the administration of Eye90 showing four structures: the rabbit’s body $B$ (yellow), the liver volume $L$ (blue), the liver extended by an isotropic 1 cm margin $L_{{{\text{shell}}}}$ (red), and the non-embolized, homogeneous background region $L_{{{\text{bkg}}}}$ (magenta)

Following the PET/CT acquisition, an additional four-phase CT was acquired with acquisition parameters set to match the parameters used during CT imaging of the calibration phantom. CT scans included a baseline unenhanced, arterial, portal, and delayed venous phase. Within MIM, the liver volume $L$ was contoured using portal phase CT to provide maximum contrast between liver parenchyma and surrounding soft tissue. The extended liver volume $L_{{{\text{shell}}}}$ was generated by isotropically extending the liver volume $L$ by a 1.0 cm margin. This margin was chosen to account for the reduced PET spatial resolution relative to CT as well as perceived ^90^Y activity outside of the liver volume $L$ resulting from respiration, which was shown during intra-procedural angiographic imaging to displace the rabbit livers by a maximum of 1.0 cm in the cranial-caudal direction. A third structure $B$ was generated around the exterior of the rabbit body. In the unenhanced CT, a planar structure *L*_bkg_ was generated in a non-embolized, homogeneous background region of the liver to account for HU variations in the non-embolized liver parenchyma between rabbits. In Fig. 2, all structures in R03 are visible in a baseline, unenhanced axial CT slice with a voxel intensity range of − 100 to 200 HU.

### Dosimetry

#### MIRD

Treatment planning for commercially available TheraSphere microspheres is based on a MIRD model that assumes a uniform ^90^Y activity distribution within a target volume [33]. In this model, the mean dose $D_{{{\text{MIRD}}}}$ to a target volume is defined in Eq. 5.

5 $$D_{{{\text{MIRD}}}} = \frac{{A_{0} \cdot 50 \cdot \left( {1 - R} \right)}}{M}$$

Here $A_{0}$ is the administered ^90^Y activity in GBq, $M$ is the mass of the target in kg, and $R$ is the fractional residual activity. To serve as a reference for PET- and CT-based dosimetry, $D_{{{\text{MIRD}}}}$ was calculated for each rabbit liver given $R$, $A_{0}$, the liver volume $L$, and an assumed liver density of 1.03 g/mL.

#### Convolution

Pathohistological studies performed on explanted human livers following ^90^Y RE have demonstrated highly heterogeneous in vivo microsphere distributions [12, 13, 34]. Currently, no clinical imaging modality can resolve individual microspheres, so all relevant imaging methods present a reduced resolution approximation of the true ^90^Y activity distribution. Within the constraints of this limitation, dose-voxel kernel (DVK) convolutional dosimetry can be used to calculate the dose distribution based on a heterogeneous ^90^Y activity distribution. In this study, the dose distribution *D* was determined through the convolution of a cumulated activity distribution $\tilde{A}$ with a spatially invariant DVK, as described in Eq. 6.

6 $$D = \tilde{A} \otimes {\text{DVK}} = \mathop \sum \limits_{{x^{\prime}}} \mathop \sum \limits_{{y^{\prime}}} \mathop \sum \limits_{{z^{\prime}}} \tilde{A}\left( {x^{\prime } ,y^{\prime } ,z^{\prime } } \right) \cdot {\text{DVK}}\left( {x - x^{\prime } ,y - y^{\prime } ,z - z^{\prime } } \right)$$

As microspheres are permanent implants, it is unnecessary to image at multiple time points post-administration to determine the cumulated activity. Therefore, $\tilde{A}$ was calculated using Eq. 7,

7 $$\tilde{A}\left( {x^{\prime } ,y^{\prime } ,z^{\prime } } \right) = \mathop \smallint \limits_{0}^{\infty } A\left( {x^{\prime } ,y^{\prime } ,z^{\prime } ,t} \right) e^{ - \lambda t} {\text{d}}t = \frac{{A\left( {x^{\prime } ,y^{\prime } ,z^{\prime } } \right)}}{\lambda } = \tau A\left( {x^{\prime } ,y^{\prime } ,z^{\prime } } \right)$$

where $\lambda$ is the decay constant, $\tau$ is the mean lifetime of ^90^Y, and $A\left( {x^{\prime } ,y^{\prime } ,z^{\prime } ,t} \right)$ is the initial ^90^Y activity in a voxel at coordinate $(x^{\prime } ,y^{\prime } ,z^{\prime } )$ at time $t$ = 0. The convolution of $\tilde{A}$ and DVK was performed in the frequency domain using the fast Fourier transform. The resulting PET- and CT-based dose distributions are subsequently referred to as ${\text{DD}}_{{{\text{PET}}}}$ and ${\text{DD}}_{{{\text{CT}}}}$, respectively. Dosimetry calculations were performed in MATLAB R2020b (MathWorks Inc., Natick, MA, USA).

#### Dose-voxel kernels

The DVKs in this study were calculated through simulations of ^90^Y radiation transport in a voxelized sphere of water with the GATE v9.0 Monte Carlo toolkit encapsulating Geant4 10.06.p01 [35, 36]. Physics processes were enabled according to option 4 of the standard electromagnetic physics list, and electron transport was performed with an energy cut-off of 1 keV. The DVKs were calculated specific to voxel sizes in the activity distributions *A*_CT_ and *A*_PET_, and are referred to as ${\text{DVK}}_{{{\text{CT}}}}$ and ${\text{DVK}}_{{{\text{PET}}}}$, respectively. Prior to each simulation, a ^90^Y source was uniformly distributed within the origin voxel of a spherical water phantom where 40 million histories were set to decay. Voxels whose centre of mass was ≤ 25 mm from the origin were assigned to water, and the remaining voxels were set to air. From the simulation output, the mean absorbed dose per history was calculated in each voxel. Voxels whose centre of mass was > 25 mm were masked to zero to ensure convolution with spherically symmetric DVKs.

#### Statistical analysis

Dosimetric evaluations were carried out through a comparison of standard dose metrics including the median dose $D_{{{\text{med}}}}$, maximum dose $D_{{{\text{max}}}}$, mean dose $D_{\mu }$, standard deviation $\sigma$, and coefficient of variation (COV) defined as $\sigma {/}D_{\mu }$. Cumulative dose-volume histograms (cDVHs) were calculated to determine $D_{70}$. The mean dose $D_{\mu }$ across all rabbits was compared between dose distributions DD_PET_ and DD_CT_ through linear regression, ANOVA, and Bland–Altman analysis. For DD_CT_, $D_{\mu }$ was calculated for the liver volume $L$. For DD_PET_, $D_{\mu }$ was calculated for the structure *L*_shell_ to account for reduced spatial resolution and respiratory motion during PET image acquisition.

## Results

### CT calibration phantom

**Fig. 3:**
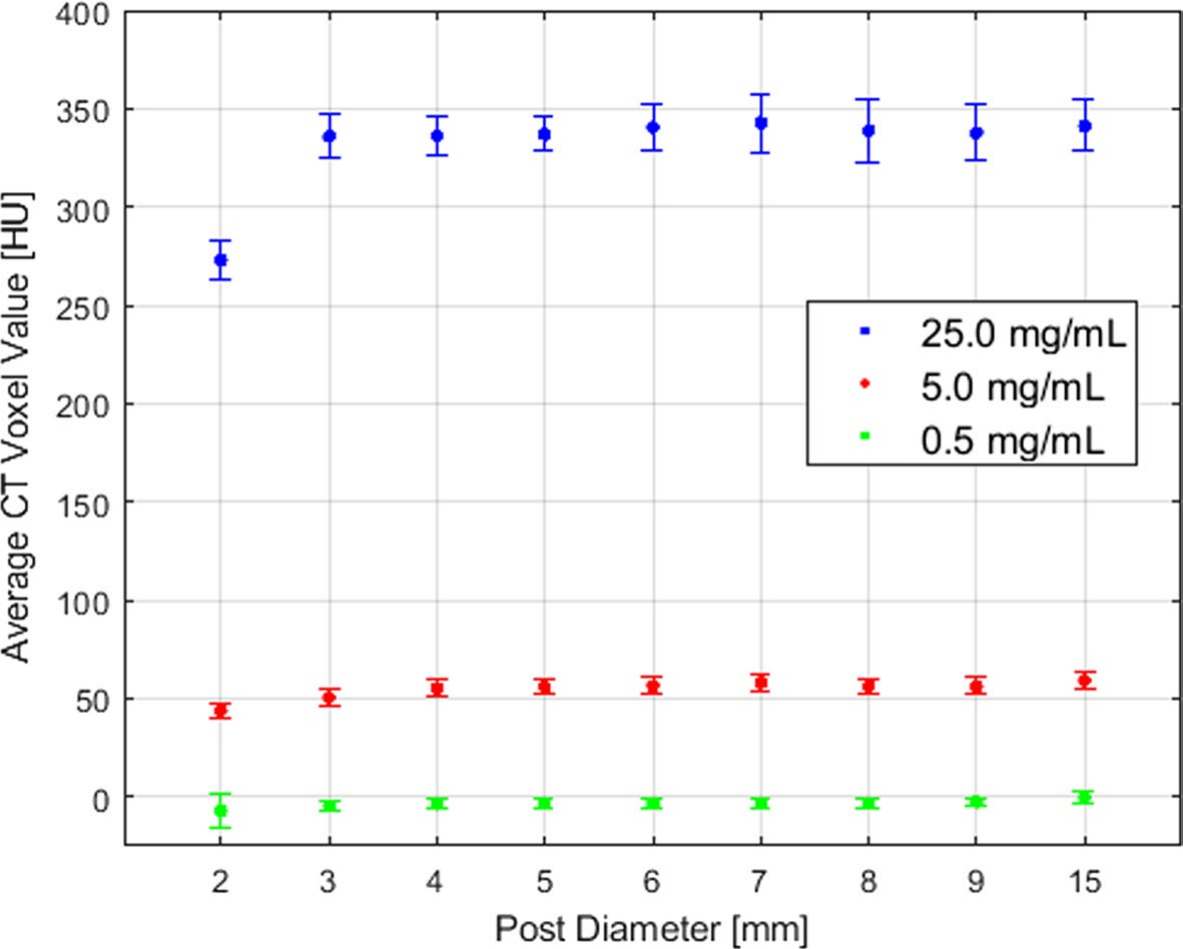
The average CT voxel value within post structures as a function of post diameter for the three microsphere concentrations within the CT calibration phantom: 0.5 mg/mL, 5.0 mg/mL, and 25.0 mg/mL. Error bars represent the standard deviation of the voxel values within a post

In Fig. 3, the mean HU is given as a function of post diameter for all three microsphere concentrations within the phantom. The data show that the mean HU is independent of the post diameter for diameters > 2 mm.

**Fig. 4:**
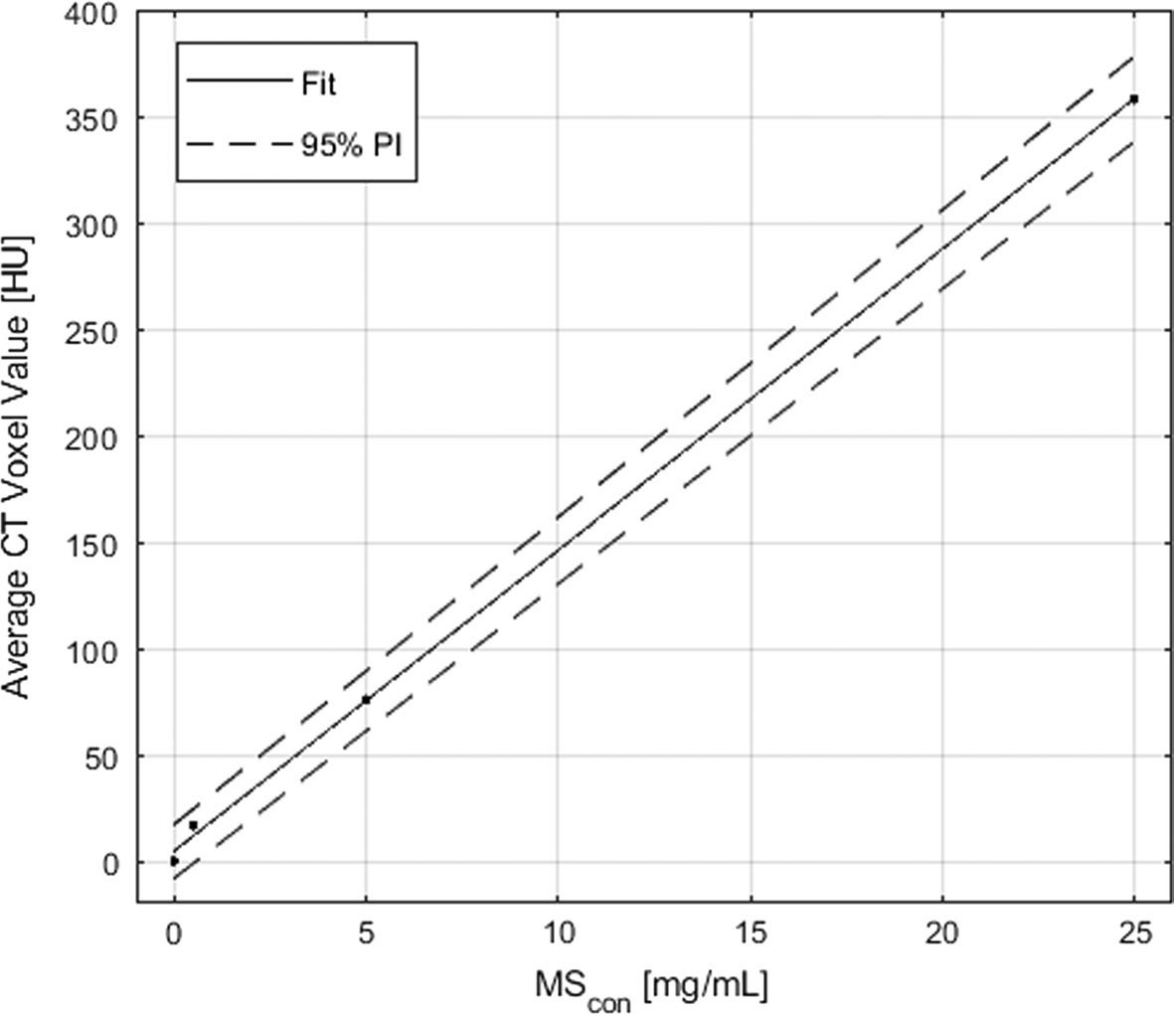
A calibration curve derived from the analysis of the calibration phantom. The coefficient of determination is *r*^2^ ≥ 0.999. Voxel values were extracted for the 15 mm-diameter posts only

The least-squares linear fit presented in Fig. 4 shows a strong correlation (*r*^2^ > 0.999) between HU and MS_con_ with upper and lower 95% prediction intervals (PIs) defined by the dashed lines. The slope is $m_{{{\text{cal}}}} = 14.13$ with 95% confidence intervals (CIs) between 13.14 and 15.12. For display purposes, data were offset such that $\mu_{{{\text{bkg}}}} = 0$.

The microsphere concentration MS_con_ within the liver volume $L$ was determined using Eq. 1 with $m_{{{\text{cal}}}} = 14.13$ and $b_{{{\text{cal}}}}$ specific to each rabbit according to Eq. 2. Rabbit liver backgrounds $\mu_{{{\text{bkg}}}}$ varied by 40.6 HU with a range of 69.2 to 109.9 HU, while $\sigma_{{{\text{bkg}}}}$ was relatively constant across all rabbits**.** Values for $\mu_{{{\text{bkg}}}}$, $\sigma_{{{\text{bkg}}}}$, and $b_{{{\text{cal}}}}$ are reported in Table 1.

**Table 1:** Average voxel values in the background region, calibration curve intercepts, activity parameters, and recovery coefficients for all rabbits

Rabbit Index	$\mu_{{{\text{bkg}}}}$ ± $\sigma_{{{\text{bkg}}}}$ (HU)	$b_{{{\text{cal}}}}$(HU)	$A_{0}$(MBq)	$A_{{{\text{CT}}}}$[95% CI] (MBq)	*A*_PET_ (MBq)	$A_{{{\text{PET}}}}^{{{\text{shell}}}}$(MBq)	${\text{RC}}_{{{\text{CT}}}}$(%)	${\text{RC}}_{{{\text{PET}}}}$(%)	${\text{RC}}_{{{\text{PET}}}}^{{{\text{shell}}}}$(%)
R01	99.1 ± 5.6	110.0	132.4	122.6 [114.6; 131.9]	93.5	138.4	92.6	70.6	104.6
R02	69.2 ± 3.0	75.1	171.0	119.8 [111.9; 128.8]	124.3	176.1	70.1	72.7	103.0
R03	84.4 ± 3.7	91.7	155.9	141.9 [132.6; 152.6]	86.5	143.2	91.1	55.5	91.9
R04	103.8 ± 4.2	112.1	128.1	117.9 [108.7; 126.8]	51.4	81.9	92.1	40.1	63.9
R05	96.5 ± 3.3	102.9	133.7	132.5 [123.8; 143.9]	99.9	144.8	99.1	74.7	108.3
R06	109.9 ± 3.8	117.2	171.0	158.2 [147.9; 171.5]	109.9	164.6	92.6	64.3	96.2
R07	105.4 ± 4.0	113.1	131.6	119.2 [110.4; 128.2]	103.3	145.0	90.6	78.5	110.2
R08	94.8 ± 4.9	104.3	130.2	100.8 [91.7; 108.4]	61.5	101.4	77.4	47.2	77.9

**Fig. 5:**
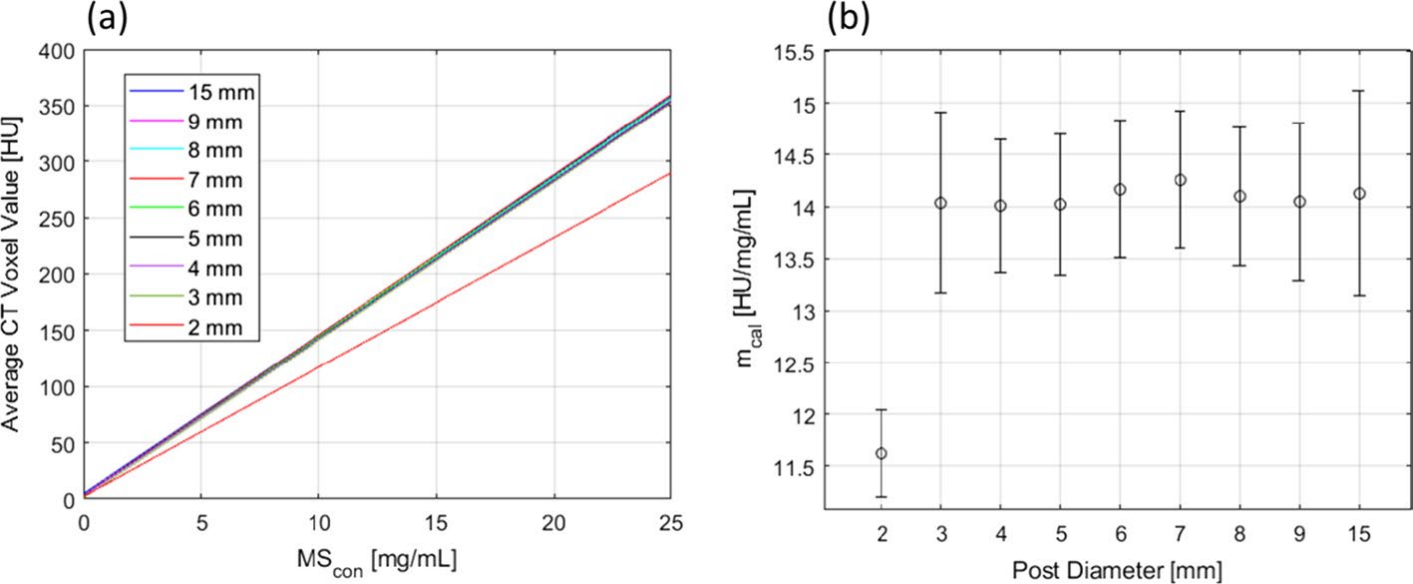
**a** Calibration curves derived for all post sizes within the calibration phantom. **b** Calibration curve slope as a function of post diameter with error bars representing 95% confidence intervals

The calibration curve used to determine the microsphere concentration within the rabbits was derived from an analysis of the 15 mm-diameter posts only, despite the presence of embolized vasculature with vessel diameters < 15 mm. An analysis of the 3 to 15 mm-diameter posts in the calibration phantom shows that $m_{{{\text{cal}}}}$ is independent of the post diameter (14.01 ≤ $m_{{{\text{cal}}}}$ ≤ 14.26) as demonstrated in Fig. 5a. The adjacent scatter plot in Fig. 5b shows that the 2 mm-diameter post ($m_{{{\text{cal}}}}$ = 11.63) has a slope outside the 95% CIs of the slopes for the remaining posts, likely due to partial volume effects.

**Fig. 6:**
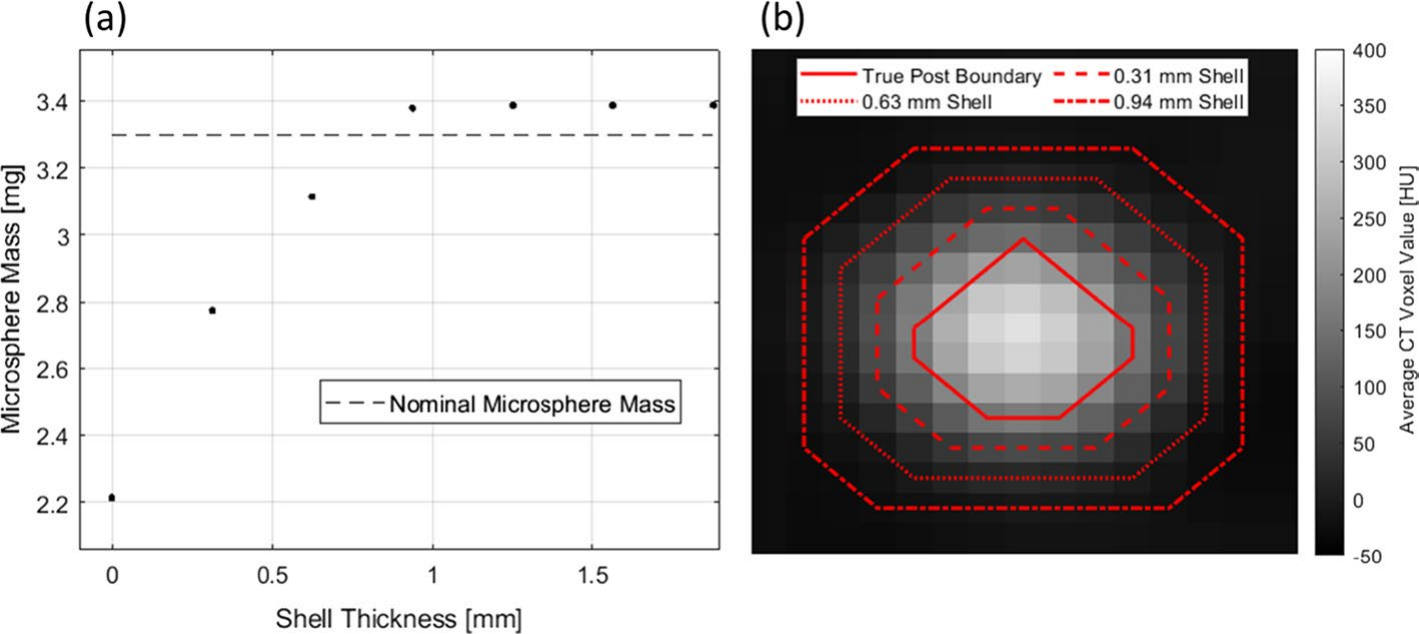
**a** The microsphere mass in the 2 mm-diameter post with a microsphere concentration of 25.0 mg/mL as a function of contour shell thickness. The nominal mass of Eye90 within the contour is represented by the dashed horizontal line. **b** Axial CT slice [− 50 to 400 HU] of the 2 mm-diameter post with a microsphere concentration of 25.0 mg/mL. The red contours represent extended shell thicknesses

To justify the use of $m_{{{\text{cal}}}}$ for vessels with diameters ≤ 2 mm, additional contours were drawn around the 2 mm-diameter post containing 25.0 mg/mL to recover the nominal mass of Eye90 within the post. Figure 6a shows the recovered mass in the post as a function of an expanding radial shell thickness up to 2 mm, and Fig. 6b shows the placement of the shells relative to the true post boundary. The data indicate that, despite the use of $m_{{{\text{cal}}}}$ derived exclusively from the 15 mm-diameter posts, the nominal mass of Eye90 within the smallest post can still be recovered within 3%.

### Activity distributions

The CT recovery coefficient ${\text{RC}}_{{{\text{CT}}}}$ was equal to 70.1% in R02 and 77.4% in R08. In the remaining six rabbits, it was found that ${\text{RC}}_{{{\text{CT}}}}$ ≥ 90.0%, while the average ${\text{RC}}_{{{\text{CT}}}}$ across all eight rabbits was 88.2 ± 8.9% [70.1; 99.1]. It is possible that the relatively low ${\text{RC}}_{{{\text{CT}}}}$ values from R02 and R08 were a result of diffuse intra- or extrahepatic Eye90 deposition where the microsphere concentration was below CT detectability limits. CT acquisitions with reduced image noise may help resolve this discrepancy. There was more variation in ${\text{RC}}_{{{\text{PET}}}}$ with an average of 62.9 ± 13.0% [40.1; 74.7], although only R02 had a PET-based activity distribution $A_{{{\text{PET}}}}$ within the 95% CIs of $A_{{{\text{CT}}}}$. Within the extended liver volume $L_{{{\text{shell}}}}$, the PET recovery coefficient ${\text{RC}}_{{{\text{PET}}}}^{{{\text{shell}}}}$ had an average of 94.5 ± 15.1% [63.9; 110.2] and R03, R06, and R08 had $A_{{{\text{PET}}}}^{{{\text{shell}}}}$ within the 95% CIs of $A_{{{\text{CT}}}}$. Activity parameters ($A_{0}$, $A_{{{\text{CT}}}}$, $A_{{{\text{PET}}}}$, $A_{{{\text{PET}}}}^{{{\text{shell}}}}$) and recovery coefficients (${\text{RC}}_{{{\text{CT}}}}$, ${\text{RC}}_{{{\text{PET}}}}$, ${\text{RC}}_{{{\text{PET}}}}^{{{\text{shell}}}}$) are reported in Table 1.

Due to the low positron fraction in ^90^Y PET imaging, scan times typically require between 15 and 30 min over which many respiratory cycles occur [37]. This effectively smears the measured ^90^Y activity distribution over a larger volume, reducing the total activity within the CT-derived liver contour. To quantify the “loss” of activity in $A_{{{\text{PET}}}}$, the ratio of ^90^Y activity outside of the extended liver volume $L_{{{\text{shell}}}}$ to the activity within the body $B$ was calculated and is reported in Table 2. On average, 14% of administered ^90^Y activity in $A_{{{\text{PET}}}}$ lies outside $L_{{{\text{shell}}}}$. A rationale for the extreme activity loss in R04 ${\text{RC}}_{{{\text{PET}}}}$ = 40.1%) is the relatively high ratio (27.0%) of ^90^Y activity beyond $L_{{{\text{shell}}}}$. The reduced spatial resolution of PET imaging relative to CT imaging may also contribute to this effect.

**Table 2:** Ratio of ^90^Y activity outside of $L_{{{\text{shell}}}}$ to the activity within $B$ to quantify extrahepatic ^90^Y activity in the PET-based activity distribution $A_{{{\text{PET}}}}$

Rabbit Index	PET activity ratio (%)
R01	10.9
R02	11.7
R03	13.7
R04	27.0
R05	10.1
R06	11.7
R07	11.9
R08	16.1

**Fig. 7:**
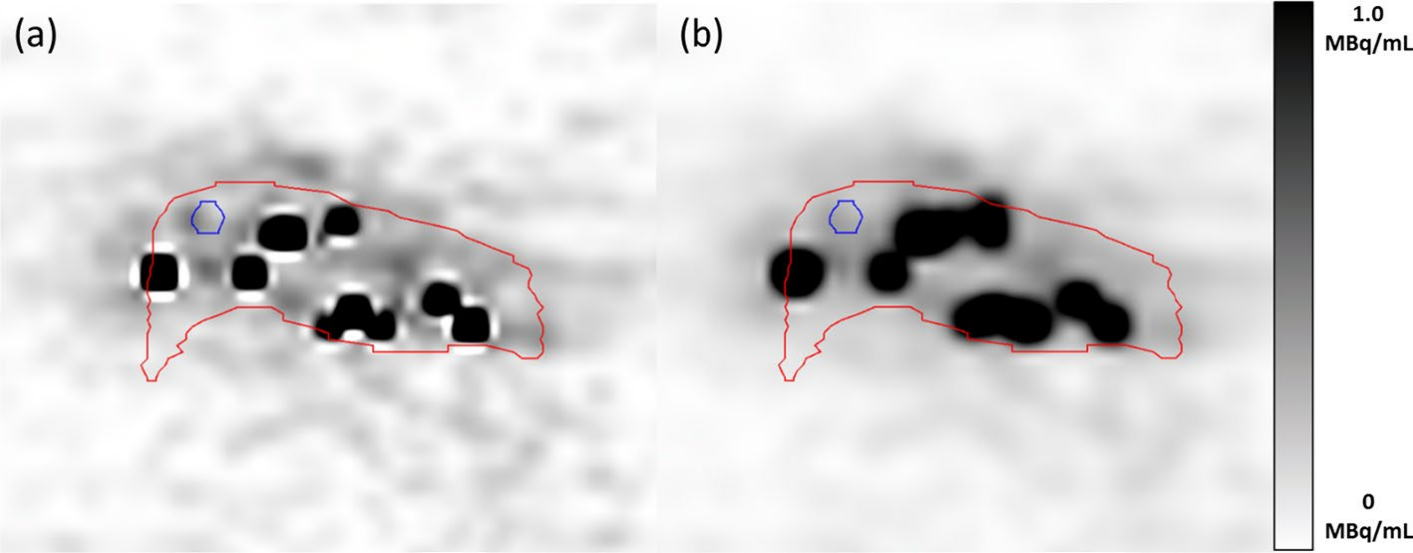
Sagittal slices of the PET-based activity distribution $A_{{{\text{PET}}}}$ **a** before filtering with a 4 mm FWHM Gaussian kernel, and **b** after filtering with a 4 mm FWHM Gaussian kernel. The red contour represents the liver volume $L$ and the blue contour represents the circular ROI where the standard deviation of ^90^Y activity concentration values was measured. The concentration values are displayed with a range of 0–1.0 MBq/mL

Post-filtering of the reconstructed ^90^Y PET images was performed to reduce image noise. Sagittal slices of $A_{{{\text{PET}}}}$ in R08 before and after applying the 4 mm Gaussian filter are provided in Fig. 7a, b, respectively. To aid in quantifying the impact of image noise, a circular region of interest (ROI) was placed in a region of low activity. Before applying the filter to $A_{{{\text{PET}}}}$, the standard deviation of ^90^Y concentration values in the ROI was 1.027 × 10^5^ Bq/mL. After filtering, the standard deviation was 4.153 × 10^4^ Bq/mL; hence, the image noise defined by the standard deviation of ^90^Y activity concentration values is a factor of ~ 2.5 greater in the unfiltered $A_{{{\text{PET}}}}$. The resulting impact is observed in the ^90^Y activity within the liver volume $L$ which is increased by 14 MBq in the unfiltered $A_{{{\text{PET}}}}$. The PET activity ratio value for R08 provided in Table 2 is reduced by 1.5% as a result of image filtering.

### Dose-voxel kernels

**Fig. 8:**
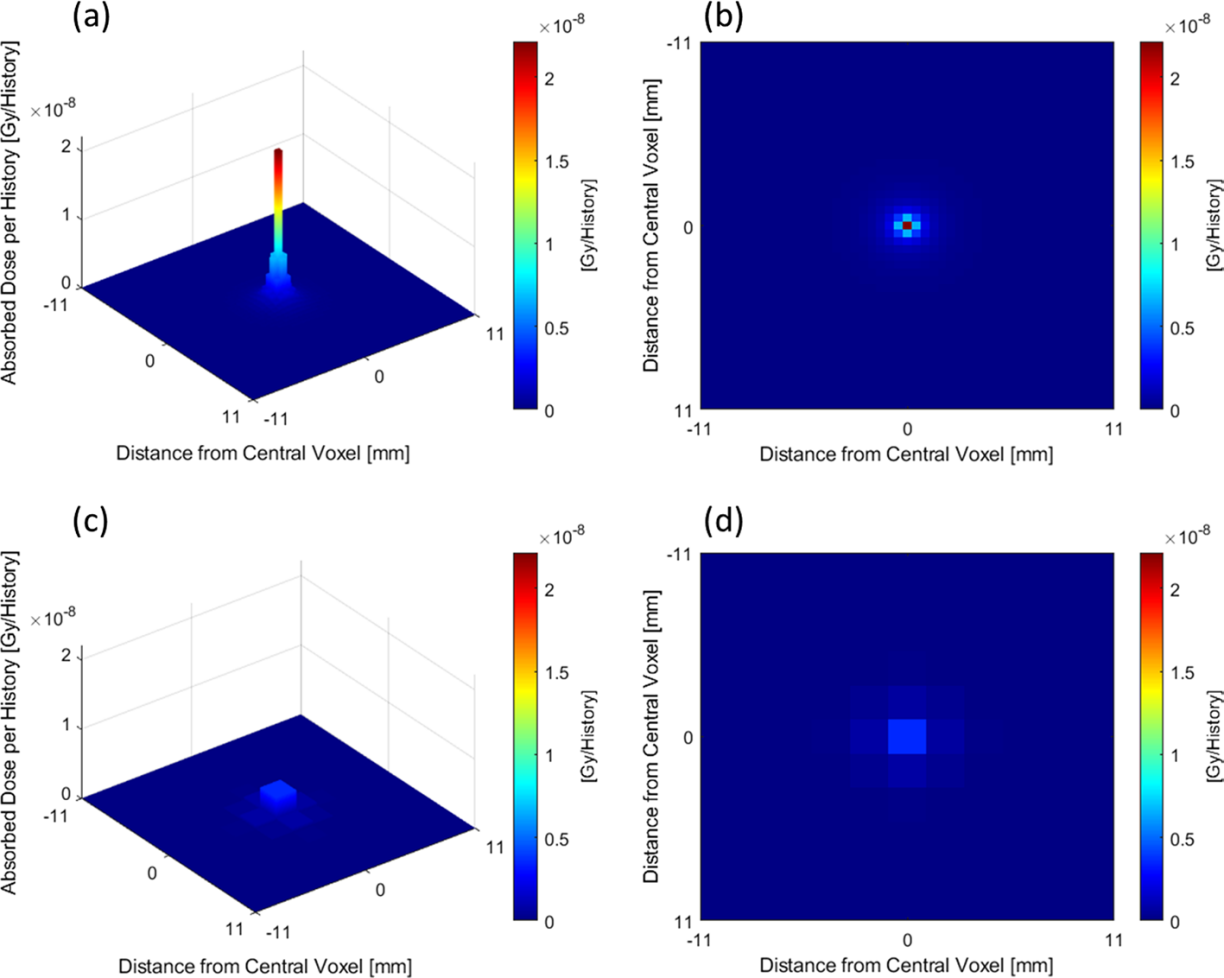
**a** 3D surface plot through the central voxel in the CT dose-voxel kernel DVK_CT_. **b** 2D cross section through the central voxel in the CT dose-voxel kernel DVK_CT_. **c** 3D surface plot through the central voxel in the PET dose-voxel kernel DVK_PET_. **d** 2D cross section through the central voxel in the PET dose-voxel kernel DVK_PET_. The maximum value in all vertical axes and colour bars corresponds to the maximum value of DVK_CT_

The absorbed dose per history in the central voxel of DVK_CT_ and DVK_PET_ was 2.21 × 10^−8^ Gy/History and 3.55 × 10^−9^ Gy/History, respectively. Both DVK_CT_ and DVK_PET_ had uncertainties ≤ 0.01% in the central voxel. A 3D surface plot and 2D cross section through the central voxel are shown for DVK_CT_ in Fig. 8a, b. The analogous plots for DVK_PET_ are shown in Fig. 8c, d. The vertical axes and colour bar limits in Fig. 8 correspond to the maximum value of DVK_CT_ in order to emphasize the difference in the magnitude of the central voxel as a result of discrete sampling.

### Dose distributions

**Fig. 9:**
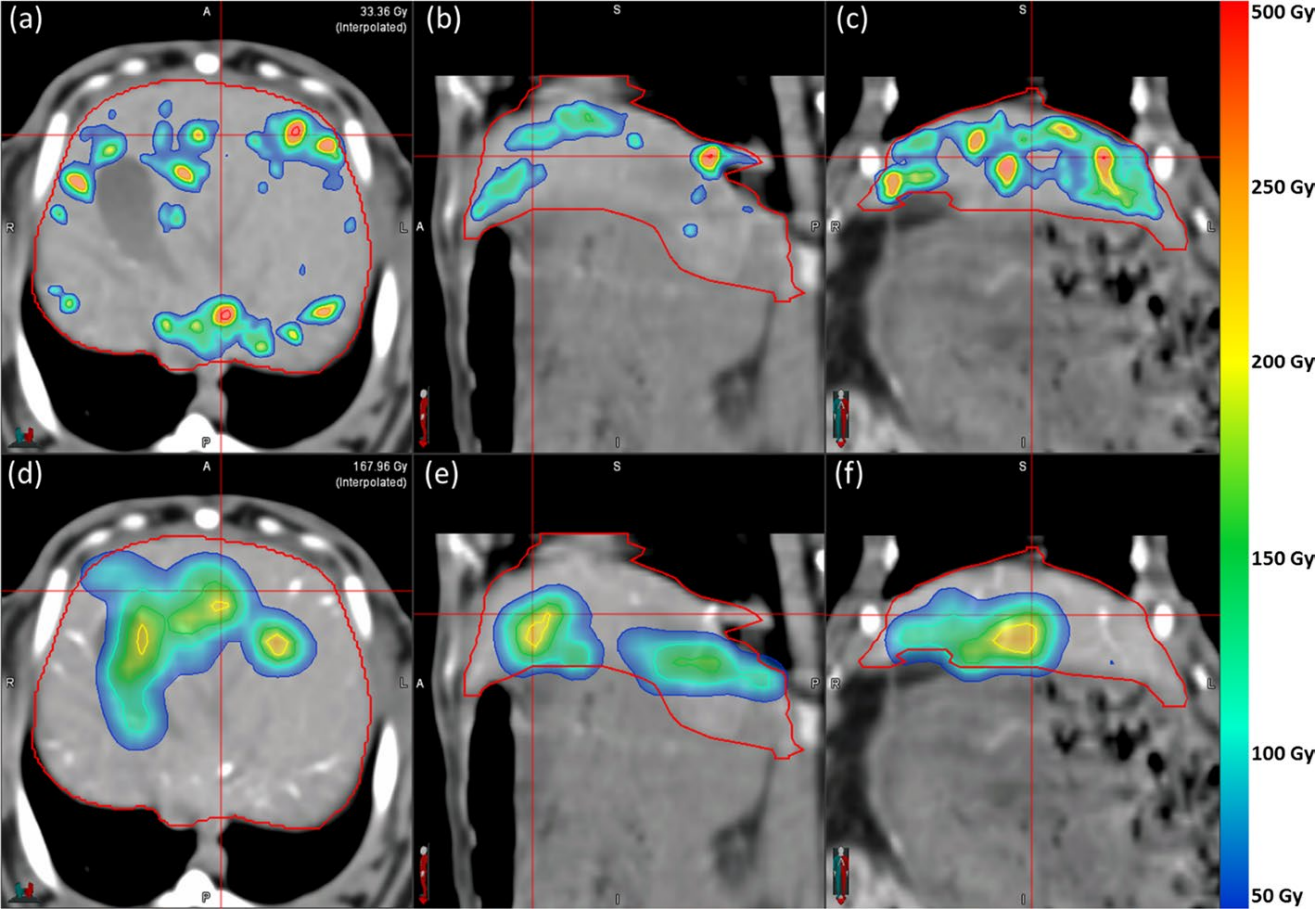
**a**–**c** Axial, sagittal, and coronal views of the CT-based dose distribution ${\text{DD}}_{{{\text{CT}}}}$ in R05 overlaid on an axial CT [− 100 to 200 HU]. **d–f** Axial, sagittal, and coronal views of the PET-based dose distribution ${\text{DD}}_{{{\text{PET}}}}$ in R05 overlaid on an axial CT [− 100 to 200 HU]

**Fig. 10:**
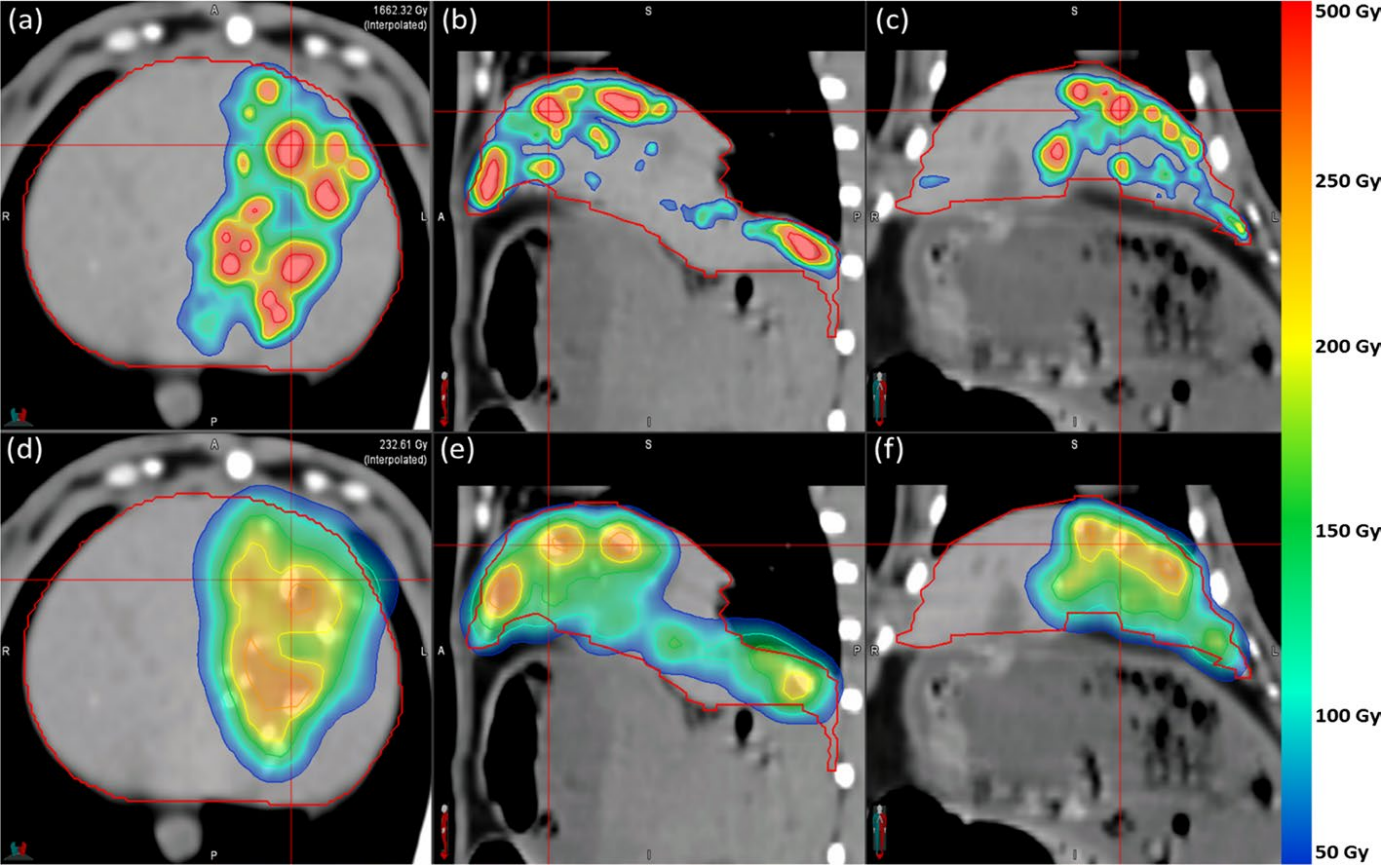
**a**–**c** Axial, sagittal, and coronal views of the CT-based dose distribution ${\text{DD}}_{{{\text{CT}}}}$ in R06 overlaid on an axial CT [-100 to 200 HU]. **d**–**f** Axial, sagittal, and coronal views of the PET-based dose distribution ${\text{DD}}_{{{\text{PET}}}}$ in R06 overlaid on an axial CT [− 100 to 200 HU]

For R05, the dose distribution DD_CT_ is shown in Fig. 9a–c and DD_PET_ is shown in Fig. 9d–f. For R06, DD_CT_ is shown in Fig. 10a–c, while DD_PET_ is shown in Fig. 10d–f. Dose distributions are overlaid on an axial CT slice with a voxel intensity range of − 100 to 200 HU. Rabbits R05 and R06 are presented as they qualitatively represent the worst and best agreement between DD_CT_ and DD_PET_. Both dose distributions are displayed with a dose range between 50 and 500 Gy.

In Fig. 9a–c, from a qualitative perspective**,** DD_CT_ appears to contain significant dose gradients and is highly correlated with the embolized vasculature as it was derived from the radiopaque microsphere distribution itself. In Fig. 9d–f, the corresponding PET-based dose distribution DD_PET_ demonstrates a clear discordance with multiple regions containing embolized vasculature. Furthermore, DD_PET_ is more homogeneous with a COV of 0.98 relative to 1.63 in DD_CT_. At the intersection of the red crosshair in Fig. 9, there is an absolute dose difference of 134.3 Gy between DD_PET_ and DD_CT_.

In Fig. 10a–f, the dose distributions ${\text{DD}}_{{{\text{PET}}}}$ and ${\text{DD}}_{{{\text{CT}}}}$ in R06 share more correspondences than in R05, although ${\text{DD}}_{{{\text{CT}}}}$ is mostly confined within the liver volume $L$, while ${\text{DD}}_{{{\text{PET}}}}$ shows dose spilling beyond $L$, thereby predicting a low dose to the surrounding soft tissue. This may be attributed to respiratory motion due to the longer scan duration in PET imaging. At the intersection of the red crosshair in Fig. 10, the absolute dose difference is 1411.2 Gy between ${\text{DD}}_{{{\text{PET}}}}$ and ${\text{DD}}_{{{\text{CT}}}}$. Quantitative dose metrics from all rabbits are reported in Table 3. As previously mentioned, the mean dose $D_{\mu }$ in ${\text{DD}}_{{{\text{CT}}}}$ was calculated for the liver volume $L$, while $D_{\mu }$ in ${\text{DD}}_{{{\text{PET}}}}$ was calculated for the extended liver volume $L_{{{\text{shell}}}}$ to account for reduced spatial resolution and respiratory motion during PET image acquisition.

**Table 3:** Dose metrics for all structures within the CT-based dose distribution ${\text{DD}}_{{{\text{CT}}}}$ and the PET-based dose distribution ${\text{DD}}_{{{\text{PET}}}}$ across all rabbits. All values are in units of Gy except for the unitless COV

Rabbit	Modality	Structure	*D*_med_	*D*_max_	*D*_*μ*_	*σ*	COV	*D*_70_
R01	CT	*L*	5.4	1124.3	42.0	78.9	1.9	0.8
		*L*_shell_	0.0	1124.3	10.9	42.9	3.9	0.4
		*B*	0.0	1124.3	3.5	24.9	7.1	0.3
	PET	*L*	36.7	372.0	65.3	66.0	1.0	11.3
		*L*_shell_	6.0	372.0	24.3	44.7	1.8	2.2
		*B*	1.6	372.0	8.9	27.3	3.1	1.1
R02	CT	*L*	9.6	1155.8	32.6	57.8	1.8	1.8
		*L*_shell_	0.0	1155.8	9.2	33.2	3.6	0.4
		*B*	0.0	1155.8	3.1	19.6	6.4	0.3
	PET	*L*	53.3	309.0	68.3	63.0	0.9	16.4
		*L*_shell_	9.3	309.1	27.3	44.4	1.6	3.7
		*B*	1.9	309.0	10.2	27.8	2.7	1.1
R03	CT	*L*	2.6	1585.5	32.5	84.7	2.6	0.9
		*L*_shell_	0.0	1585.5	10.5	48.2	4.6	0.4
		*B*	0.0	1585.5	4.5	31.8	7.2	0.3
	PET	*L*	15.9	345.0	42.0	55.0	1.3	10.3
		*L*_shell_	8.2	345.0	21.3	37.7	1.8	5.3
		*B*	3.1	345.0	10.4	25.8	2.5	2.0
R04	CT	*L*	14.3	754.8	41.9	68.3	1.6	4.3
		*L*_shell_	0.0	754.8	11.3	39.0	3.4	0.2
		*B*	0.0	754.8	3.9	23.5	6.0	0.1
	PET	*L*	23.4	266.0	36.9	36.3	1.0	15.4
		*L*_shell_	8.5	266.0	15.8	23.8	1.5	6.1
		*B*	3.7	266.0	7.4	15.0	2.0	2.0
R05	CT	*L*	2.9	1812.3	38.4	96.3	2.5	0.8
		*L*_shell_	0.1	1812.3	16.4	64.3	3.9	0.4
		*B*	0.0	1812.3	3.7	31.0	8.4	0.3
	PET	*L*	21.7	389.0	58.5	66.0	1.1	9.4
		*L*_shell_	6.5	389.3	22.7	43.0	1.9	2.1
		*B*	1.6	389.0	9.0	27.1	3.0	1.0
R06	CT	*L*	13.6	1375.6	49.7	91.2	1.8	3.3
		*L*_shell_	0.0	1375.6	13.5	50.5	3.7	0.5
		*B*	0.0	1375.6	4.4	29.7	6.7	0.3
	PET	*L*	67.5	337.0	72.0	54.2	0.8	39.3
		*L*_shell_	9.9	336.8	28.4	41.3	1.5	4.6
		*B*	1.9	337.0	10.5	26.2	2.5	1.0
R07	CT	*L*	6.1	1005.1	30.8	63.2	2.0	1.1
		*L*_shell_	0.0	1005.1	9.1	36.1	4.0	0.2
		B	0.0	1005.1	3.2	21.9	6.8	0.2
	PET	L	30.6	316.0	54.5	55.0	1.0	12.4
		L_shell_	6.5	316.4	22.1	38.5	1.7	3.4
		*B*	1.9	316.0	8.8	24.5	2.8	1.2
R08	CT	*L*	11.8	966.2	30.2	49.1	1.6	4.0
		*L*_shell_	0.1	966.2	8.3	27.7	3.3	0.2
		*B*	0.0	966.2	3.5	18.5	5.3	0.2
	PET	*L*	22.5	304.0	39.9	41.2	1.0	11.8
		*L*_shell_	7.7	304.4	16.7	27.2	1.6	5.0
		*B*	3.2	304.0	8.4	18.6	2.2	2.0

**Fig. 11:**
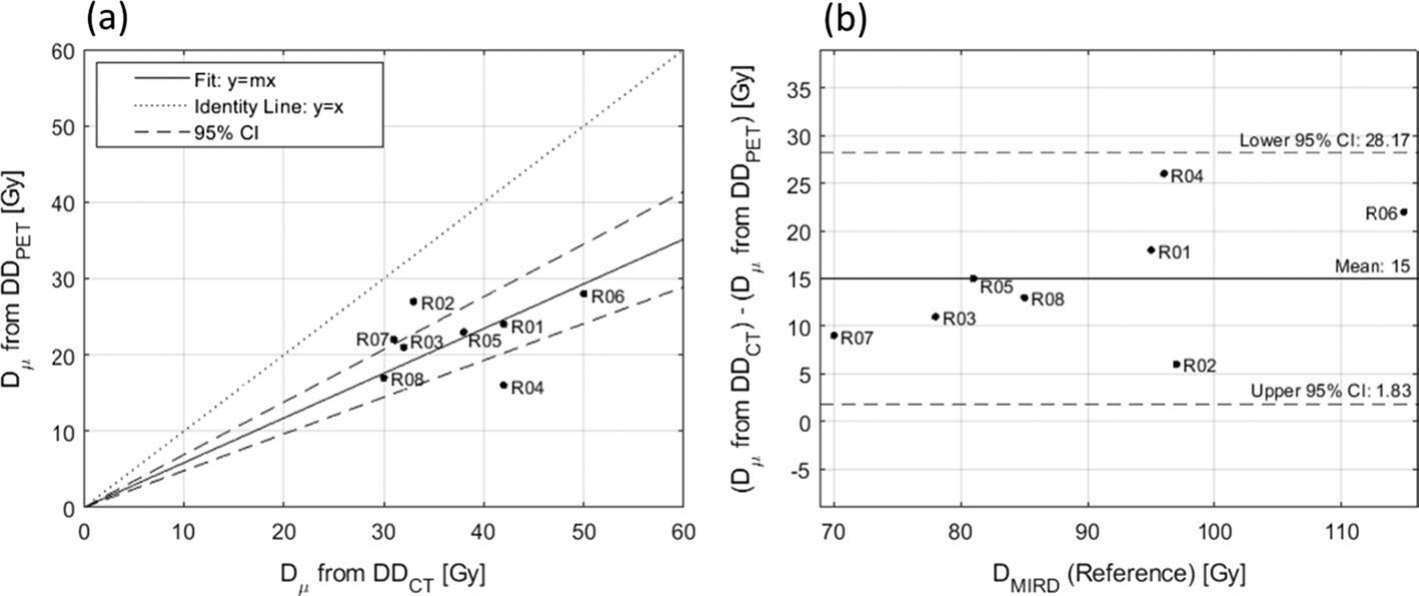
**a** Linear regression analysis with 95% confidence intervals (dashed) and identity line (dotted) for the mean dose $D_{\mu }$ in the CT-based dose distribution ${\text{DD}}_{{{\text{CT}}}}$ and in the PET-based dose distribution ${\text{DD}}_{{{\text{PET}}}}$. **b** Bland–Altman analysis with 95% confidence intervals (dashed)

The results from linear regression are shown in Fig. 11a, and results from Bland–Altman analysis are shown in Fig. 11b. Linear regression reveals a poor correlation with a coefficient of determination of $r^{2} = 0.374$ between the mean dose $D_{\mu }$ in ${\text{DD}}_{{{\text{CT}}}}$ and in ${\text{DD}}_{{{\text{PET}}}}$. The slope of the curve is 0.586 with 95% CIs between 0.482 and 0.693. Bland–Altman analysis predicts a mean offset of 15.0 Gy between $D_{\mu }$ in ${\text{DD}}_{{{\text{CT}}}}$ and in ${\text{DD}}_{{{\text{PET}}}}$ with 95% CIs between 1.8 Gy and 28.2 Gy.

**Fig. 12:**
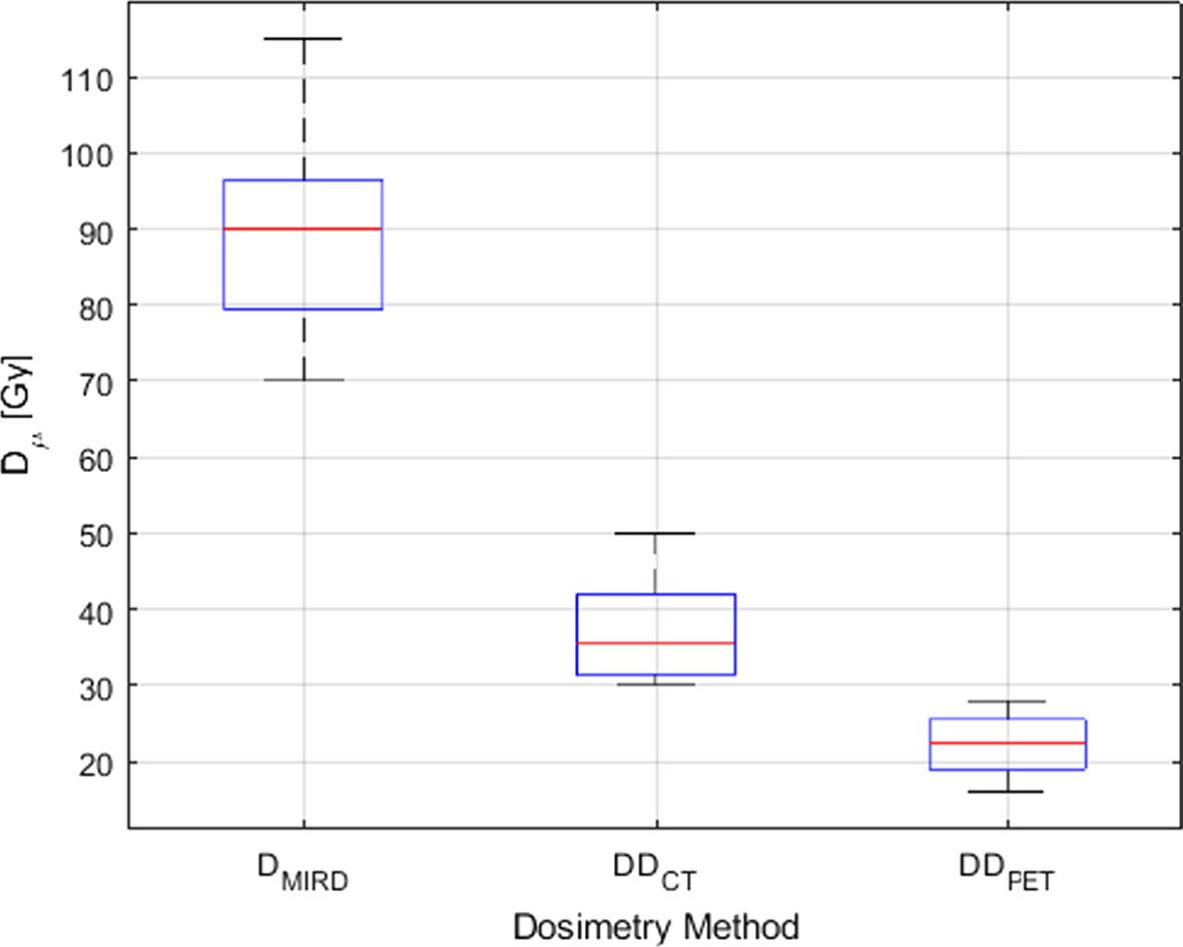
Box-and-whisker plot for the mean dose $D_{\mu }$ across all rabbits extracted from the CT-based dose distribution ${\text{DD}}_{{{\text{CT}}}}$, the PET-based dose distribution ${\text{DD}}_{{{\text{PET}}}}$, and ${\text{DD}}_{{{\text{MRD}}}}$. The red line represents the median value, the blue box contains data between the 25th and 75th percentiles, and the black whiskers extend to the most extreme data points

Figure 12 shows a box-and-whisker plot of the mean dose $$D_{\mu }$$ from $${\text{DD}}_{{{\text{CT}}}}$$ and $${\text{DD}}_{{{\text{PET}}}}$$, as well as the MIRD-derived dose $${\text{DD}}_{{{\text{MRD}}}}$$ The data indicate a larger dispersion in $$D_{\mu }$$ for $${\text{DD}}_{{{\text{CT}}}}$$ relative to $${\text{DD}}_{{{\text{PET}}}}$$. Median values for PET, CT and MIRD are 22.5 Gy, 35.5 Gy, and 90.0 Gy, respectively, with interquartile ranges between 19.0 and 25.5 Gy, 31.5 and 42.0 Gy, and 79.5 and 96.5 Gy, respectively. Results from ANOVA indicate $${\text{DD}}_{{{\text{MRD}}}}$$ and $$D_{\mu }$$ from $${\text{DD}}_{{{\text{CT}}}}$$ and $${\text{DD}}_{{{\text{PET}}}}$$ are all significantly different with $$F\left( {2,21} \right) = 113.2$$, $$p = 5.65 {\text{x}} 10^{ - 12}$$. If the absorbed dose is averaged over every constituent voxel of the dose matrices $${\text{DD}}_{{{\text{CT}}}}$$ and $${\text{DD}}_{{{\text{PET}}}}$$, the discrepancy in $$D_{\mu }$$ is resolved. In this case, the average difference in $$D_{\mu }$$ between $${\text{DD}}_{{{\text{CT}}}}$$ and $${\text{DD}}_{{{\text{PET}}}}$$$${\text{DD}}_{{{\text{PET}}}}$$ is only 1.6 ± 0.2 Gy [1.3; 2.0].

### Dose-volume histograms

**Fig. 13:**
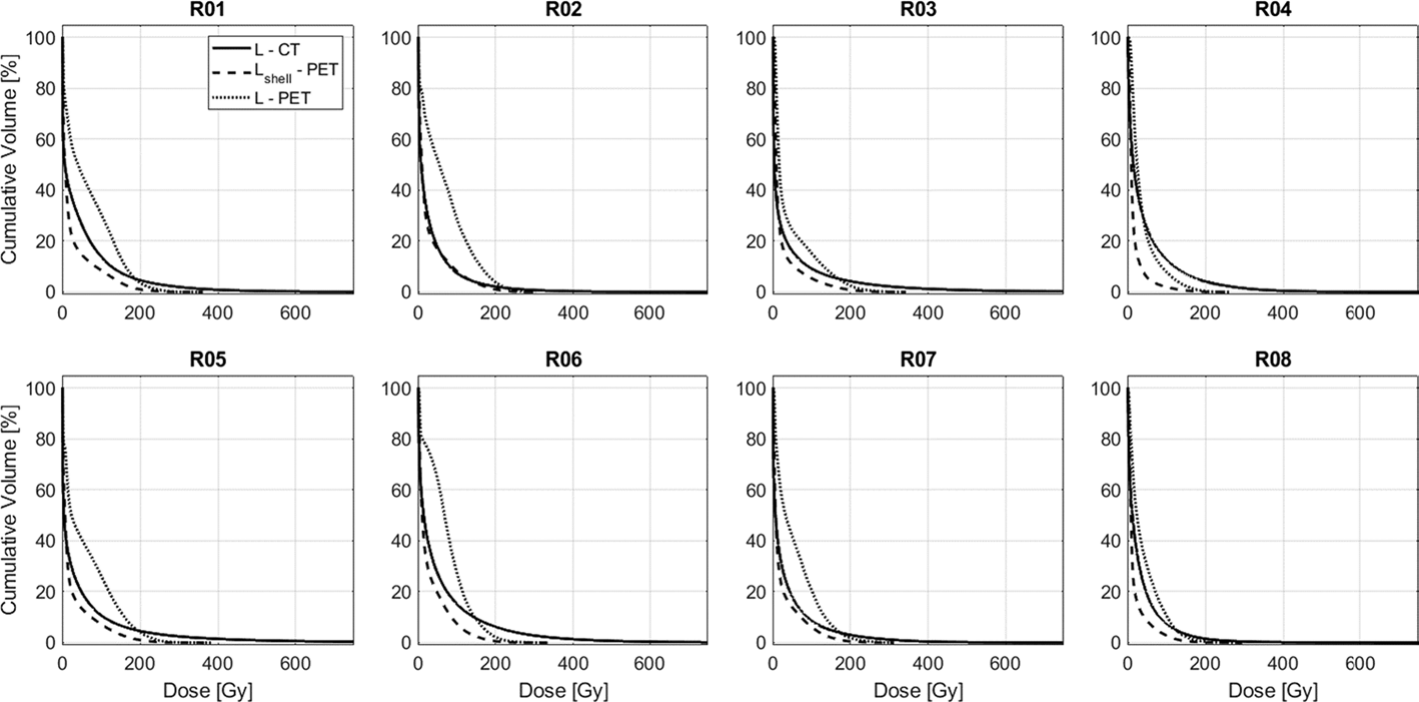
Cumulative dose volume histograms (cDVHs) for the liver volumes $L$ and $L_{{{\text{shell}}}}$ across all rabbits

Shown in Fig. 13 are cDVHs in the liver volumes $L$ and $L_{{{\text{shell}}}}$ for all rabbits. The broader shoulder of the cDVH curves for ${\text{DD}}_{{{\text{PET}}}}$ indicates a bias toward lower doses relative to ${\text{DD}}_{{{\text{CT}}}}$. The long tail of the liver cDVH derived from ${\text{DD}}_{{{\text{CT}}}}$ implies that a small fraction of the liver volume $L$ received an exceedingly high dose, with the highest dose in R05 greater than 1800 Gy. However, maxima never exceed 389 Gy in ${\text{DD}}_{{{\text{PET}}}}$. In most cases, there is better agreement in the cDVH curves of $L$ for ${\text{DD}}_{{{\text{CT}}}}$ and $L_{{{\text{shell}}}}$ for ${\text{DD}}_{{{\text{PET}}}}$.

## Discussion

### CT calibration phantom

Figure 6 suggests that diffusion of HU enhancement to surrounding voxels, in part due to CT image acquisition and reconstruction parameters, may introduce additional blurring of the dose over small distance scales (recall 100% of the microsphere mass is recovered within a 0.85 mm-thick shell beyond the 2 mm-diameter most), but the total microsphere quantification could be highly accurate. In Figs. 9 and 10, many high intensity regions are clearly larger in cross sectional area than the equivalent area of the 2 mm-diameter post, further suggesting the impact of these partial volume effects may not be clinically consequential. This can be verified by the similar values for recovery coefficients ${\text{RC}}_{{{\text{CT}}}}$ and ${\text{RC}}_{{{\text{PET}}}}^{{{\text{shell}}}}$ reported in Table 1. An experimental calibration phantom featuring conical inserts that taper to a point is under development to further explore the dependence of the calibration curve slope on diameters ≤ 2 mm.

### Alternative calibration method

An alternative method in the development of CT calibration curves would require pre- and post-CT imaging of a liver tumour before and after microsphere administration to determine a relative HU enhancement, ΔHU. The application of this alternative calibration method could address existing complications in the current calibration scheme. For example, the contour $L_{bkg}$ was defined in a region void of microspheres despite the microsphere bolus being administered, in some cases, to the whole liver via the proper hepatic artery. Therefore, the structure $L_{bkg}$ had to be placed carefully to avoid embolized vasculature. This subjective placement of $L_{bkg}$ is a limitation in this study that could potentially contribute to dosimetric uncertainty. The advantage of the ΔHU calibration method is that it would remove the necessity, and associated uncertainty, of defining $L_{bkg}$. Unfortunately, the ΔHU calibration method was not applied in this work as respiratory motion artefacts produced undesirable results following image co-registration with the post-treatment CT. Human patients, however, can perform breath holds during their pre- and post-treatment CT to reduce these image discrepancies.

The ΔHU calibration method requires a post-treatment CT scan, and therefore results in additional radiation dose to the patient. The average effective dose for an abdominal CT is estimated to be 8 mSv, which is several orders of magnitude less than the absorbed dose received during a standard ^90^Y RE procedure [38]. Given the poor 5-year survival rate of primary liver cancer (20%), the risk of carcinogenesis resulting from a routine abdominal CT scan is negligible [39]. This logic also applies to liver metastases arising from the most common sites (colorectal, neuroendocrine, and breast tumours) given their poor 5-year survival rates of 11%, 54%, and 23%, respectively [40–43]. Although it is important to optimize the CT imaging protocol to minimize the absorbed radiation dose to patients, in this case, low dose imaging will increase image noise and propagate to uncertainty in ^90^Y absorbed dose estimates. Physicians must select CT imaging protocols to balance the competing objectives of performing accurate dosimetry and reducing CT imaging dose, though the importance of accurate dosimetry in the therapeutic procedure may be of paramount importance. Iterative image reconstruction techniques could be selected to improve CT image quality with lower dose acquisitions.

### Minimum detectable activity

We applied the ΔHU calibration approach to the calibration phantom in an effort to determine a theoretical limit of detection ${\text{LOD}}_{{{\text{HU}}}}$ as described by the Clinical and Laboratory Standards Institute [44] and previously implemented in determining the detectable iodine concentrations within anthropomorphic phantoms [45]. If the population of voxels within the background is normally distributed with a mean voxel value $\mu_{{{\text{bkg}}\_{\text{phant}}}} = 0$ (following the ΔHU calibration method) and a standard deviation $\sigma_{{{\text{bkg}}\_{\text{phant}}}}$, the limit of blank ${\text{LOB}}_{{{\text{HU}}}}$ is the voxel value found when replicates of $\mu_{{{\text{bkg}}\_{\text{phant}}}}$ are repeatedly measured. CT voxels with HU < ${\text{LOB}}_{{{\text{HU}}}}$ have a 95% probability of not containing Eye90 microspheres. The ${\text{LOB}}_{{{\text{HU}}}}$ is defined in Eq. 8.

8 $${\text{LOB}}_{{{\text{HU}}}} = \mu_{{{\text{bkg}}\_{\text{phant}}}} + 1.645\sigma_{{{\text{bkg}}\_{\text{phant}}}}$$

The ${\text{LOD}}_{{{\text{HU}}}}$ is the lowest microsphere concentration to be reliably distinguished from the ${\text{LOB}}_{{{\text{HU}}}}$ and is determined by measuring replicates of a sample known to contain a low microsphere concentration (0.5 mg/mL), as defined in Eq. 9.

9 $${\text{LOD}}_{{{\text{HU}}}} = {\text{LOB}}_{{{\text{HU}}}} + 1.645\sigma_{0.5}$$

We applied the calibration curve parameters to define ${\text{LOD}}_{{{\text{HU}}}}$ in terms of microsphere concentration ${\text{LOD}}_{{\text{mg/mL}}}$ using Eq. 10.

10 $${\text{LOD}}_{{\text{mg/mL}}} = \frac{{{\text{LOD}}_{{{\text{HU}}}} - b_{{{\text{cal}}}} }}{{m_{{{\text{cal}}}} }}$$

The minimum detectable ^90^Y activity concentration from CT imaging ${\text{MDA}}_{{{\text{CT}}}}$ was determined from ${\text{MS}}_{{{\text{mg}}}}$, $A_{{{\text{MS}}}}$, and ${\text{LOD}}_{{\text{mg/mL}}}$, as shown in Eq. 11.

11 $${\text{MDA}}_{{{\text{CT}}}} = {\text{LOD}}_{{\text{mg/mL}}} \cdot {\text{MS}}_{{{\text{mg}}}} \cdot A_{{{\text{MS}}}}$$

Given ${\text{MS}}_{{{\text{mg}}}}$ = 24,460 MS/mg, the average microsphere specific activity of $A_{{{\text{MS}}}}$ = 156 Bq, and ${\text{LOD}}_{{\text{mg/mL}}}$ = 0.22 mg/mL, Eq. 11 yields ${\text{MDA}}_{{{\text{CT}}}}$≈ 0.84 MBq/mL. The corresponding value in TOF ^90^Y PET imaging has previously been estimated as ${\text{MDA}}_{{{\text{PET}}}}$ = 1.0 MBq/mL for small lesions [46]. We applied Eq. 5 to determine the corresponding dose in a voxel volume given $A_{0} = {\text{MDA}}_{{{\text{CT}}}} \cdot V_{{{\text{CT}}}}$, *M* = 1.03 g/mL·*V*_CT_, and *R* = 0. For a single PET voxel, the MIRD dose corresponding to ${\text{MDA}}_{{{\text{PET}}}}$ was 49 Gy, while the MIRD dose for a single CT voxel was 40 Gy. These threshold dose values justify the lower limit of the dose distribution colour bar in Figs. 9 and 10 and are also well below the estimated dose threshold required to produce a therapeutic response. This implies that CT is not inferior to PET in terms of detectable activity and dose levels while still being capable of producing more realistic heterogeneous dose distributions. The value of ${\text{MDA}}_{{{\text{CT}}}}$ also suggests that CT-based dosimetry will be sensitive enough to characterize regions of the target volume that may be at risk for disease progression. Accurately assessing the tumour coverage is essential as low dose regions correlate with reduced local control and an increased probability of local recurrence [47].

In a clinical ^90^Y microsphere administration, several GBq of ^90^Y activity are administered to a target volume, depending on the treatment planning approach [48]. In this study, the average administered activity was only 144 MBq due to the desire to achieve clinically relevant dose levels in the perfused liver volume. PET and CT quantification accuracy can be expected to improve when delivering additional activity (to increase the dose delivered or to perfuse larger territories), which would increase the PET signal and image contrast. Moreover, the presence of a hypervascular tumour in this animal model may further concentrate a larger fraction of the administered ^90^Y activity into a tissue volume that is significantly smaller than the whole liver. This would likely have significant implications for the ^90^Y activity heterogeneity in both the tumour and normal liver anatomy. The effect of increased administered ^90^Y activities and the presence of hypervascular tumour models will need to be investigated in future clinical studies.

In this study, a microsphere mass of 40 mg (~ 981,000 microspheres) and ^90^Y activities between 140 and 180 MBq were chosen to provide clinically relevant dose levels on the order of 120 Gy. The average microsphere specific activity, at the time of administration, was calculated as 156 Bq. This value is expected to increase for Eye90 administration in human patients where larger target volumes are encountered. For reference, TheraSphere microspheres are calibrated at a specific activity of 2,500 Bq, although this value may be significantly lower at the time of administration when accounting for ^90^Y decay due to the 12-day shelf life of the product [49]. The administered number of TheraSphere microspheres is in the range of 1.2 to 8 million [33]. SIR-spheres, however, are calibrated at a much lower microsphere specific activity between 50 and 150 Bq while administered numbers of microspheres are on the order of tens of millions [50].

### Dose discrepancies

Figure 12 reveals significant differences between $D_{{{\text{MIRD}}}} , \;{\text{DD}}_{{{\text{CT}}}}$, and ${\text{DD}}_{{{\text{PET}}}}$. A rationale for the large value of $D_{{{\text{MIRD}}}}$ relative to the mean dose in ${\text{DD}}_{{{\text{CT}}}}$, and ${\text{DD}}_{{{\text{PET}}}}$ lies in an assumption of the MIRD model—the ^90^Y activity is fully contained and uniformly distributed within the target volume [33]. In ^90^Y RE, the clinical reality is that microspheres lodge in vascular regions most often found in the periphery of the tumour [13]. This results in absorbed dose spilling outside of the tumour contour due to the ^90^Y β^−^ range. In this study, 37.2 ± 5.2% [27.5; 43.8] of the CT-based ^90^Y activity lies within 2.4 mm (mean range of ^90^Y β^−^ particle) of the exterior boundary of $L$ and 99.2 ± 1.4% [95.8; 100.0] of the ^90^Y activity lies within 11.0 mm (maximum range of ^90^Y β^−^ particle) of the exterior boundary of $L$. The discrepancy between $D_{{{\text{MIRD}}}} , \;{\text{DD}}_{{{\text{CT}}}}$, and ${\text{DD}}_{{{\text{PET}}}}$ is expected to be reduced in human patients having larger liver volumes. Dose discrepancies observed between ${\text{DD}}_{{{\text{CT}}}}$ and ${\text{DD}}_{{{\text{PET}}}}$ are attributed to imaging modality-specific characteristics as described in the following sections.

#### PET

The chief limitations in ^90^Y PET imaging result from the low positron fraction (~ 32 ppm). At very low counts, PET images are noisy, and the resulting scatter correction may lead to a significant under or overestimation of the scatter contribution [51]. Moreover, bremsstrahlung photons and prompt gammas can result in a very high fraction of random coincidences when imaging ^90^Y. Corrections for scatter and randoms generate noise in the true coincidence sinogram of count-deprived images, with the potential for a large fraction of negative counts. Reconstruction algorithm positivity constraints truncate negative values which can cause an overestimation of ^90^Y activity due to a positive bias in low or zero activity regions in the reconstructed image [51].

An additional limitation lies in the choice of scintillator material. Most modern TOF PET scanners utilize lutetium-based scintillators, such as cerium-doped lutetium oxyorthosilicate LSO(Ce) or lutetium-yttrium oxyorthosilicate LYSO(Ce). Although these have desirable characteristics in terms of temporal resolution, light output, and detection efficiency, the presence of naturally occurring radioisotope lutetium-176 produces undesirable intrinsic background counts within the scintillator that can reduce quantitative accuracy in the case of low counts and a high randoms fraction [52].

A fundamental physical constraint in PET imaging that may contribute to the differences between ${\text{DD}}_{{{\text{PET}}}}$ and ${\text{DD}}_{{{\text{CT}}}}$ is the positron range and non-collinearity of the annihilation photons. The electron–positron pair produced in the decay of ^90^Y shares a maximum kinetic energy of 0.739 MeV, corresponding to a root mean square positron range of ~ 0.5 mm [53, 54]. Depending on the residual energy of the positron at the time of annihilation, the annihilation photons may not be emitted exactly colinearly. These effects contribute additional uncertainty in determining the true ^90^Y activity distribution.

In this study, given the range of liver motion observed during intra-procedural angiographic imaging (1.0 cm), there may be a degree of dosimetric uncertainty as a result of PET/CT co-registration. A study by Vogel et al. reported 40% of patients encounter an absolute PET/CT co-registration error exceeding 1 cm in the cranial-caudal dimension when employing an expiration breath hold technique during CT imaging [55]. The same study found CT-based attenuation correction artefacts caused a deformation of the liver dome in the PET image > 1 cm in 50% of patients. Based on these results, deviations in the mean dose $D_{\mu }$ can be expected between ${\text{DD}}_{{{\text{PET}}}}$ and ${\text{DD}}_{{{\text{CT}}}}$, particularly for tumours proximal to the liver dome. An advantage of CT-based dosimetry in ^90^Y RE is the absence of the requirement of image co-registration between PET and CT—a necessity for accurate anatomical localization and CT-based attenuation corrections. Therefore, CT-based dosimetry is less prone to dosimetric uncertainty resulting from voluntary or involuntary patient motion.

Respiratory motion is thought to be responsible for the low ${\text{RC}}_{{{\text{PET}}}}$ values in Table 1 as the ^90^Y activity is effectively smeared over a larger volume during respiration. This results in the projection of some fraction of the activity into voxels that lie outside of the liver contour $L$ defined by the post-treatment CT scan. By including an additional shell around the liver ($L_{{{\text{shell}}}}$), much of the activity lost in ${\text{RC}}_{{{\text{PET}}}}$ is recovered in ${\text{RC}}_{{{\text{PET}}}}^{{{\text{shell}}}}$.

#### CT

An issue specific to post-treatment CT imaging in ^90^Y RE is the use of vascular contrast agents when guiding the catheter through the hepatic arterial vasculature. Although a rabbit-specific calibration curve intercept $b_{{{\text{cal}}}}$ accounts for varying mean background intensity $\mu_{{{\text{bkg}}}}$, non-uniform contrast agent uptake within the liver volume $L$ was still observed. In these cases, $\mu_{{{\text{bkg}}}}$ may be sensitive to the spatial location of $L_{{{\text{bkg}}}}$, so it is essential to place multiple $L_{{{\text{bkg}}}}$ structures within $L$ to average over any nonuniformities.

An additional drawback in implementing a CT-based approach to dosimetry in ^90^Y RE is that it involves an indirect method of ^90^Y activity quantification that requires the intermediate step of imaging and analysing a calibration phantom. This may introduce additional uncertainty in ${\text{DD}}_{{{\text{CT}}}}$. For example, the calibration curve slope $m_{{{\text{cal}}}}$ was calculated with 95% CIs. In R05, when using the upper 95% CI in $m_{{{\text{cal}}}}$ to determine ${\text{DD}}_{{{\text{CT}}}}$, $D_{\mu }$ decreased by 2.5 Gy, from 38.6 Gy to 35.9 Gy. When using the lower 95% CI in $m_{{{\text{cal}}}}$, $D_{\mu }$ increased by 3.3 Gy, from 38.6 Gy to 41.7 Gy. The corresponding *A*_CT_ values for the upper and lower 95% CIs were 123.8 MBq and 143.9 MBq, respectively, while $A_{0}$ was 133.7 MBq.

CT image artefacts may present additional complications. High-Z objects proximal to the intended treatment target, such as clips, coils, or calcifications, could produce image artefacts that may be mistaken for radiopaque microsphere uptake. In these cases, image artefacts could be mitigated by implementing the ΔHU calibration approach discussed previously. Note the Eye90 in this study did not produce any image artefacts as a result of its material composition.

Hypervascular tumours receiving considerable amounts of Eye90 are expected to demonstrate significant CT voxel enhancement. The HCC criteria outlined in the modified response evaluation criteria in solid tumours (mRECIST) rely on measurements of perfused volumes following the administration of contrast agents in follow-up CT imaging [56]. When implementing mRECIST, there is potential uncertainty in defining the true source of CT voxel enhancement, whether it be from Eye90 or a vascular contrast agent. However, in mRECIST evaluation, there is a baseline non-contrast scan, and therefore, the presence of any residual Eye90 will be known and can potentially be accounted for by the radiologist during mRECIST assessment. The impact of permanently implanted radiopacity on image-based assessments of tumour response (e.g. mRECIST) will need to be studied in the future.

### Dose heterogeneity

This study has demonstrated a significant benefit of CT-based dosimetry is its ability to reveal the high dose heterogeneity known to exist from pathohistological studies on explanted livers following ^90^Y RE [12, 13]. The COV values in Table 3 verified this heterogeneity as the COV within the liver volume $L$ in ${\text{DD}}_{{{\text{CT}}}}$ was consistently greater than the corresponding values in $DD_{PET}$ with an average of 1.99 ± 0.35 [1.63; 2.61] in ${\text{DD}}_{{{\text{CT}}}}$ and 1.02 ± 0.15 [0.75; 1.31] in ${\text{DD}}_{{{\text{PET}}}}$. The high spatial resolution of CT also provides an opportunity to further investigate dose-volume metrics for predicting response in ^90^Y RE. One of the earliest studies reporting dose-volume metrics in ^90^Y RE was performed by a Kao et al. where $D_{70}$ > 100 Gy was suggested as a threshold to predict treatment response in HCC [57]. A subsequent study by Fowler et al. indicated dose-volume metrics predict response better in hypovascular lesions than in hypervascular ones, and suggested one incorporate a measure of tumour dose heterogeneity, such as the COV, into the dose response analysis to improve the positive predictive value [58]. Willowson et al. later found $D_{70}$ resulted in a stronger correlation with outcome than $D_{\mu }$ in metastatic colorectal cancer patients [59]. More recently, a study performed by Kappadath et al. found $D_{\mu }$ and $D_{20}$ to $D_{80}$ were correlated with mRECIST response criteria [60]. We have previously shown that $D_{70}$ is dependent on the spatial resolution of the imaging modality (26), and in this study, the data showed $D_{70}$ in the liver volume $L$ (and in $L_{{{\text{shell}}}}$) were consistently overestimated in ${\text{DD}}_{{{\text{PET}}}}$ relative to the corresponding values in ${\text{DD}}_{{{\text{CT}}}}$. Although these results were not derived from a hypervascular tumour model, they suggest that existing dose–response data based on dose-volume metrics derived from PET and bSPECT may be improved with CT-based dosimetry. The potential for ^90^Y CT-based dosimetry to refine our understanding of the dose–response relationship in ^90^Y RE should be evaluated in future clinical studies.

## Conclusions

The recovery of the radiopaque microsphere mass within the rabbits validates CT-based dosimetry in ^90^Y RE. Due to the high resolution of CT imaging, the benefits of this novel approach include improved visualization of the dose distribution, reduced partial volume effects in dose reporting, and a better representation of dose heterogeneity allowing for the extraction of more accurate dose-volume metrics. Effects of respiratory motion are also mitigated when compared to post-treatment PET or bSPECT imaging. Future work aims to validate these benefits in a hypervascular tumour model, and to use larger sample sizes to validate our confidence in implementing CT-based dosimetry in ^90^Y RE. In summary, post-treatment CT imaging of radiopaque microspheres provides the means to perform precision dosimetry in ^90^Y RE, extract accurate dose metrics used to refine the understanding of the dose–response relationship, and ultimately improve future patient outcomes.

## Data Availability

The datasets analysed during the current study are available from the corresponding author on reasonable request.

## Abbreviations

^90^Y: Yttrium-90
cDVH: Cumulative dose-volume histogram
CI: Confidence interval
COV: Coefficient of variation
CSDA: Continuous slowing down approximation
CT: Computed tomography
DVK: Dose-Voxel Kernel
FOV: Field of view
FWHM: Full width at half maximum
HCC: Hepatocellular carcinoma
HU: Hounsfield units
LOB: Limit of blank
LOD: Limit of detection
MAA: Macro-aggregated albumin
MDA: Minimum detectable activity
MIRD: Medical internal radiation dose
mRECIST: Modified response evaluation criteria in solid tumours
PET: Positron emission tomography
PI: Prediction interval
RE: Radioembolization
ROI: Region of interest
SPECT: Single photon emission computed tomography
TOF: Time of flight
