# Correction: Precision dosimetry in yttrium-90 radioembolization through CT imaging of radiopaque microspheres in a rabbit liver model

**DOI:** 10.1186/s40658-023-00596-x

**Published:** 2023-11-29

**Authors:** E. Courtney Henry, Matthew Strugari, George Mawko, Kimberly Brewer, David Liu, Andrew C. Gordon, Jeffrey N. Bryan, Charles Maitz, James J. Karnia, Robert Abraham, S. Cheenu Kappadath, Alasdair Syme

**Affiliations:** 1 Department of Physics and Atmospheric Science, Dalhousie University, Halifax, NS Canada; 2 Biomedical Translational Imaging Centre, Halifax, NS Canada; 3 Department of Medical Physics, Nova Scotia Health Authority, Halifax, NS Canada; 4 Department of Radiation Oncology, Dalhousie University, Halifax, NS Canada; 5 Department of Diagnostic Radiology, Dalhousie University, Halifax, NS Canada; 6 Department of Biomedical Engineering, Dalhousie University, Halifax, NS Canada; 7 ABK Biomedical Inc., Halifax, NS Canada; 8 School of Biomedical Engineering, University of British Columbia, Vancouver, BC Canada; 9 Department of Radiology, Northwestern University, Chicago, IL USA; 10 Department of Veterinary Medicine and Surgery, University of Missouri, Columbia, MO USA; 11 Department of Imaging Physics, University of Texas MD Anderson Cancer Centre, Houston, TX USA

## Correction: EJNMMI Phys 9, 21 (2022)  https://doi.org/0.1186/s40658-022-00447-1

In this article [1], James J. Karnia at affiliation “Department of Veterinary Medicine and Surgery, University of Missouri, Columbia, MO, USA” was missing from and has been added to the author list.

The Authors’ Contributions section has been revised to:

ECH performed the data analysis and wrote the manuscript. MS and KB calculated and validated the dose-voxel kernels. DL provided editorial review. JNB and ACG contributed to the protocol development, execution of the experiments, and interpretation of the data. CM performed structure delineation in rabbit CT volumes. JJK performed the interventional procedures. RJA contributed to the protocol development, data analysis, and interpretation of the data. SCK contributed to the data analysis and interpretation of the data. AS and GM contributed to the conceptualization and review of the study. All authors contributed to the drafting and revision of the manuscript. All authors read and approved the final manuscript.

The original article [1] has been updated.
